# Pathogenic *LRRK2* variants are gain-of-function mutations that enhance LRRK2-mediated repression of β-catenin signaling

**DOI:** 10.1186/s13024-017-0153-4

**Published:** 2017-01-19

**Authors:** Daniel C. Berwick, Behzad Javaheri, Andrea Wetzel, Mark Hopkinson, Jonathon Nixon-Abell, Simone Grannò, Andrew A. Pitsillides, Kirsten Harvey

**Affiliations:** 1 Department of Pharmacology, UCL School of Pharmacy, University College London, 29-39 Brunswick Square, London, WC1N 1AX UK; 2 Department of Health, Life and Chemical Sciences, The Open University, Walton Hall, Milton Keynes, MK6 7AA UK; 3 Department of Comparative Biomedical Sciences, The Royal Veterinary College, Royal College Street, London, NW1 0TU UK

**Keywords:** LRRK2, β-catenin, Parkinson’s disease, Wnt signaling, Osteoporosis

## Abstract

**Background:**

*LRRK2* mutations and risk variants increase susceptibility to inherited and idiopathic Parkinson’s disease, while recent studies have identified potential protective variants. This, and the fact that LRRK2 mutation carriers develop symptoms and brain pathology almost indistinguishable from idiopathic Parkinson’s disease, has led to enormous interest in this protein. LRRK2 has been implicated in a range of cellular events, but key among them is canonical Wnt signalling, which results in increased levels of transcriptionally active β-catenin. This pathway is critical for the development and survival of the midbrain dopaminergic neurones typically lost in Parkinson’s disease.

**Methods:**

Here we use *Lrrk2* knockout mice and fibroblasts to investigate the effect of loss of Lrrk2 on canonical Wnt signalling in vitro and in vivo. Micro-computed tomography was used to study predicted tibial strength, while pulldown assays were employed to measure brain β-catenin levels. A combination of luciferase assays, immunofluorescence and co-immunoprecipitation were performed to measure canonical Wnt activity and investigate the relationship between LRRK2 and β-catenin. TOPflash assays are also used to study the effects of LRRK2 kinase inhibition and pathogenic and protective *LRRK2* mutations on Wnt signalling. Data were tested by Analysis of Variance.

**Results:**

Loss of Lrrk2 causes a dose-dependent increase in the levels of transcriptionally active β-catenin in the brain, and alters tibial bone architecture, decreasing the predicted risk of fracture. *Lrrk2* knockout cells display increased TOPflash and *Axin*2 promoter activities, both basally and following Wnt activation. Consistently, over-expressed LRRK2 was found to bind β-catenin and repress TOPflash activation. Some pathogenic LRRK2 mutations and risk variants further suppressed TOPflash, whereas the protective R1398H variant increased Wnt signalling activity. LRRK2 kinase inhibitors affected canonical Wnt signalling differently due to off-targeting; however, specific LRRK2 inhibition reduced canonical Wnt signalling similarly to pathogenic mutations.

**Conclusions:**

Loss of LRRK2 causes increased canonical Wnt activity in vitro and in vivo*.* In agreement, over-expressed LRRK2 binds and represses β-catenin, suggesting LRRK2 may act as part of the β-catenin destruction complex. Since some pathogenic *LRRK2* mutations enhance this effect while the protective R1398H variant relieves it, our data strengthen the notion that decreased canonical Wnt activity is central to Parkinson’s disease pathogenesis.

**Electronic supplementary material:**

The online version of this article (doi:10.1186/s13024-017-0153-4) contains supplementary material, which is available to authorized users.

## Background

Parkinson’s disease (PD) is an incurable progressive movement disorder that is characterized by the degeneration of dopaminergic neurons of the *substantia nigra pars compacta*. PD is the second most common neurodegenerative disease worldwide [1–3]. Although typically idiopathic, genetic studies have identified a strong hereditary contribution to PD risk. Interest in PD-causing mutations in the *LRRK2* gene is particularly strong, since *LRRK2* mutations account for up to 40% of PD cases in some populations, and elicit symptoms and brain pathologies resembling idiopathic PD [1–3]. As such, uncovering the function of *LRRK2* is expected to be hugely informative for understanding early PD aetiology and developing novel treatments for this condition.

*LRRK2* encodes leucine-rich repeat kinase 2 (LRRK2), a 2527 amino acid protein that has been implicated in the regulation of various cellular functions, including autophagy and endocytosis. LRRK2 contains two distinct enzymatic activities, namely serine/threonine kinase activity and GTPase activity, the latter conferred by a RocCOR (Ras of complex proteins; C-terminal of Roc) tandem domain. The combination of these enzymatic activities suggests a function for LRRK2 in signal transduction [1, 2, 4–6]. LRRK2 has been implicated in the regulation of a number of signal transduction pathways, for example JNK [7], FAS [8], NFAT [9], and NF-κB [10]. In addition, LRRK2 has been reported to be phosphorylated by IκK kinases in response to Toll-like receptor activation [11], and by casein kinase 1α [12]. However, a definitive, conserved cellular role for LRRK2 has yet to emerge, suggesting that it may serve distinct functions in different cell types [6].

Another signal transduction cascade linked to LRRK2 is canonical Wnt signalling [13–19]. Wnt (Wingless/Int) pathways are a family of evolutionarily conserved signal transduction cascades best described in developmental biology and cancer [20–22]. Activation of the canonical Wnt pathway, induces the nuclear accumulation of the transcriptional co-factor β-catenin, with resultant changes in gene expression [20–22]. In the absence of stimulus, β-catenin is repressed by retention in a multi-protein β-catenin destruction complex. Here, β-catenin is phosphorylated by glycogen synthase kinase-3β (GSK3β) triggering its continual ubiquitination and degradation [20–22].

Perturbed canonical Wnt signalling has been suggested to underlie a variety of clinical conditions. Increased Wnt activity is well established in the causation of many types of cancer, most notably cancers of the bowel [21], whilst decreased Wnt signalling is heavily involved in melanoma [23]. Alterations in Wnt signalling are also implicated in kidney disease [24], pulmonary and hepatic fibroses [25, 26], and a number of neurological conditions, including Alzheimer’s disease, Schizophrenia, depression, and Parkinson’s disease [27–29]. The above list notwithstanding, the bodily tissue that appears most exquisitely sensitive to changes in Wnt signalling is bone. In both humans and mice, increased Wnt signalling has been shown to cause increased bone strength and, in severe cases, osteopetrosis [30–35]. Conversely, decreased Wnt signalling leads to weakened bones and osteoporosis [30, 36–41].

We previously reported a role for LRRK2 as a scaffold protein in canonical Wnt signalling [16]. Via direct interaction with the co-receptor LRP6 [16], dishevelled (DVL) proteins [14] and GSK3β [15], LRRK2 assists in the formation of signalosomes following activation of the canonical Wnt pathway. Interestingly, the strength of interactions between LRRK2 and LRP6, DVLs and GSK3β are all affected by *LRRK2* mutations [14–16] and consistent with this, the extent to which over-expressed LRRK2 is able to augment DVL1-driven canonical Wnt activation was reduced by the pathogenic R1441C, Y1699C and G2019S *LRRK2* mutations [16]. The *LRRK2* PD risk variant, G2385R, behaves similarly in these assays [18]. Most notably, the protective *LRRK2* variant, R1398H, has the opposite effect, actually enhancing DVL1-driven β-catenin activation [18]. Curiously however, despite our data supporting a positive role for LRRK2 in *activated* Wnt signalling, siRNA-mediated knockdown of this protein also led to enhanced Wnt activation, both under basal and stimulated conditions [16]. We hypothesized that this represented an additional role for LRRK2 in basal Wnt signalling, as a component of the β-catenin destruction complex.

Here, we provide compelling evidence that the primary role of LRRK2 in the canonical Wnt pathway is as a repressor of β-catenin. Fibroblasts derived from LRRK2 knockout mice display enhanced canonical Wnt activity compared to wild-type controls, whilst over-expressed LRRK2 inhibits transcription driven by exogenous β-catenin. Most importantly, aged LRRK2 knockout mice display increased predicted tibial bone strength as well as increased free β-catenin levels in brain. Finally, we present evidence that *LRRK2* mutations are inhibitory to canonical Wnt signalling under tonic conditions, while the protective R1398H variant enhances this pathway. Thus, our data strengthen the growing connection between LRRK2, PD and the canonical Wnt pathway.

## Methods

### Mice

B6.129X1(FVB)-*Lrrk2* ^*tm1.1Cai*^/J (“*Lrrk2* knockout”) animals, containing a targeted deletion of exon 2 of the *Lrrk2* gene [42], were obtained from Jackson Labs and housed at the UCL School of Pharmacy. *Lrrk2* knockout mice were bred against a C57BL/6J background and genotyped according to recommended protocols. All procedures followed UK Home Office approval.

### High-resolution micro-computed tomography (μ-CT)

Tibiae from 60 to 64 week old (*n* = 7/age group) *Lrrk2* knockout and wild-type female mice were fixed in 4% paraformaldehyde and stored in 70% EtOH until scanning using the Skyscan 1172 (Skyscan, Kontich, Belgium), with the x-ray tube operated at 50 kV and 200 μA, 1600 ms exposure time with a 0.5 mm aluminium filter and a voxel size of 5 μm. The scanning time for each sample was approximately 2 h. The slices were then reconstructed using NRecon 1.6.9.4 (Skyscan, Kontich, Belgium). 2D/3D analyses were performed using CTAn 1.13.5.1+ version software (Skyscan, Kontich, Belgium). Finally, CTvox 2.7.0 r990 version (Skyscan, Kontich, Belgium) was used for 3D visualization [43].

### Morphometrical analysis

#### Trabecular analysis

Prior to analysis, μ-CT images were re-oriented in DataViewer 1.5.0 (Skyscan, Kontich, Belgium), such that the cross-section within the transverse plane was perpendicular to the long axis of the bone. Tibial length was measured in CTAn 1.13.5.1+ software using a straight line measuring tool and the appearance of the trabecular ‘bridge’ connecting the two primary spongiosa bone ‘islands’ was set as reference point for analysis of the metaphyseal trabecular bone adjacent to the epiphyseal growth plate. 5% of the total bone length from this point (towards the diaphysis) was utilised for trabecular analysis of the proximal tibia. The selected trabecular regions of interests were analysed using CTAn BatMan software (Skyscan, Kontich, Belgium) and morphometric parameters were recorded.

#### Whole bone cortical analysis

Whole bone analysis was performed on datasets derived from whole CT scans using BoneJ (version 1.13.14) [44] a plugin for ImageJ [45]. Following segmentation, alignment and removal of fibula from the dataset, a minimum bone threshold was selected for each bone to separate higher density bone from soft tissues and air. The most proximal and the most distal 10% portions of tibial length were excluded from analysis, as these regions include trabecular bone. This threshold was used in “Slice Geometry” within BoneJ to calculate cross sectional area (CSA), second moment of area around the minor axis (Imin), second moment of area around the major axis (Imax), mean thickness determined by local thickness in two dimensions (Ct.Th), Ellipticity, resistance to torsion (J) and section modulus around the major (Zmax) and minor (Zmin) axes.

### Mouse brain biochemistry

#### Preparation of protein extracts

Brains taken from 22 to 26 week old male or 60–64 week old female mice were rinsed in phosphate-buffered saline (PBS) and immediately transferred to ice. All subsequent steps were performed at 4 °C. Brains were homogenized into 5 ml brain lysis buffer [50 mM Tris, pH 7.5, 150 mM NaCl, 5 mM MgCl_2_ and 1% (*v/v*) NP-40, supplemented with 1× complete protease inhibitor cocktail (Roche) and 1× Halt phosphatase inhibitor cocktail (Pierce)] using a dounce homogenizer and then clarified by centrifugation at 14 000 *g* for 10 min. Samples for Western blotting were denatured by the addition of 4× LDS sample loading buffer and 10× sample reducing agent (both Invitrogen) followed by heating to 99 °C for 10 min. Samples for ECT pulldown experiments were assayed for protein concentration by bicinchoninic acid (BCA) assay (Pierce).

#### ECT pulldowns of Free β-Catenin

Active β-catenin was pulled-down from lysates using a protocol derived from that described by Luckert and colleagues [46]. Briefly, brains were extracted as above and a volume equivalent to 2 mg total protein taken from each lysate and added to 20 μl GST-ECT beads. Pulldowns were performed by incubating beads and lysates overnight at 4 °C on a rotor, to allow the binding of monomeric β-catenin to ECT protein. The next morning, GST-ECT beads were washed five times in brain lysis buffer before protein complexes were denatured by the addition of 4× LDS sample loading buffer and 10× sample reducing agent (both Invitrogen) followed by heating to 99 °C for 10 min.

### Generation of GST-ECT beads

To induce expression of GST-ECT protein, BL21 *Escherichia coli* cells transformed with pETM33-ECT were grown to an OD600 of 0.5 and treated with 100 μM (final concentration) Isopropyl β-D-1-thiogalactopyranoside (IPTG), prior to culture at 30 °C for 5 h. Cells were pelleted by centrifugation and pellets washed twice with ice-cold PBS. Bacterial pellets were resuspended in 25 ml of bacterial lysis buffer [20 mM Tris (pH 7.5), 150 mM NaCl, 5 mM MgCl_2_, 2 mM EDTA (pH 8), 1% (*v/v*) Triton X-100, 1X complete protease inhibitor cocktail (Roche), 2 mM dithiothreitol, 0.25 mg/ml lysozyme (Sigma) and 10 μg/ml DNAseI (Sigma)] and subjected to at least three freeze-thaw cycles. Extracts were clarified by centrifugation and transferred to a fresh tube, with a small aliquot used to verify protein expression by Coomassie staining. In parallel, 1 ml of Glutathione Sepharose 4 Fast Flow beads (GE Healthcare) was washed in 5 ml bacterial lysis buffer. To generate GST-ECT beads, the beads were added to cleared bacterial lysate and incubated for 30 min at 4 °C on a rotator. Beads were washed five times in bacterial lysis buffer and resuspended in the same buffer supplemented with 50% glycerol, prior to storage at −20 °C. GST-ECT beads were equilibrated into brain lysis buffer before use in ECT pulldown experiments.

### Expression constructs

pcDNA3-FLAG-β-catenin (Addgene plasmid #16828; [47]), Axin2-luciferase (Addgene plasmid #21275; [48]), V405 HA-CK1ε (wild-type) and V406 HA-CK1ε (K38R) (Addgene plasmids #13724 and #13725; [49]), and HA GSK3 beta wt pcDNA3 (Addgene plasmid #14753; [50]) were gifts from Eric Fearon, Frank Costantini, David Virshup, and Jim Woodgett respectively. pCMV5-HA-AXIN1 was obtained from MRC PPU Reagents. Mammalian expression plasmids encoding myc-tagged LRRK2 with N1437H and R1628P mutations were made using site-directed mutagenesis to pRK5-mycLRRK2 [14]. Plasmids were verified by DNA sequencing. pETM33-ECT was made by PCR amplification of DNA encoding amino acids 732 to the end of E-cadherin protein from human brain cDNA, follow by in-frame insertion into the NcoI and SalI site of the bacterial expression plasmid, pETM-33. Primers: forward: ctgtccatggggaggagagcggtggtcaaagag; reverse: ctcgcagctgctagtggtcctcgccgcctccg. All other constructs have been described previously [14, 16, 18, 51].

### Cell culture and Transfection

HEK293 cells (ATCC CRL1573) and LRRK2 knockout and wild-type control mouse embryonic fibroblast cells [51] were grown in Dulbecco’s modified Eagle’s medium (DMEM) supplemented with 10% (*v/v*) foetal bovine serum, 2 mM glutamine, 100 U/ml penicillin G and 100 μg/ml streptomycin at 37 °C in 95% air-5% CO_2_. Cells for immunofluorescence were seeded onto poly-d-lysine-coated coverslips. In all cases cells were transfected with Fugene HD reagent (Roche) and harvested 24 h post-transfection.

### Inhibitor treatments

The LRRK2 kinase inhibitors LRRK2-in-1 [52], TAE684 [53] and CZC25146 [54] were kindly provided by the Michael J. Fox Foundation. All compounds were resuspended in DMSO, with serial dilutions also made using DMSO. Cells were transferred into media containing inhibitors at the indicated concentrations (or DMSO only) 6 h after transfection and incubated for 20 h prior to lysis. The final DMSO concentration was fixed at 0.1% (*v/v*).

### Co-Immunoprecipitation

Cells were grown in 10 cm plates and transfected with 4 μg of the relevant plasmids. Twenty-four hours later cells were lysed in a solution containing 150 mM NaCl, 50 mM Tris (pH 7.5), 2 mM EDTA (pH 8), 1% (*v/v*) Triton X-100, plus 1X complete protease inhibitor cocktail (Roche) and 1X Halt phosphatase inhibitor cocktail. Following centrifugation (4 °C, 15 min, 16,000 *g*) 1 ml of cell lysate was added to 40 μl of anti-FLAG M2 affinity gel (Sigma) and incubated for 1 h at 4 °C on a turning disk. The affinity gel was washed four times in extraction buffer and FLAG fusion proteins eluted with 150 ng of 3 × FLAG peptide (Sigma) for 30 min at room temperature. Eluates were denatured by the addition of 4× LDS sample loading buffer and 10× sample reducing agent (both Invitrogen) followed by heating to 99 °C for 10 min.

### Western blotting

Protein samples were loaded onto 4–12% (*w/v*) BisTris pre-cast gels (Invitrogen). Proteins were transferred to polyvinylidine fluoride (PVDF) membranes (Millipore), rinsed briefly in Tris-buffered saline plus 0.1% (*v/v*) Tween 20 (TBS-T), and blocked by incubation in TBS-T containing 5% (*w/v*) non-fat dry milk (blocking buffer) for 30 min. Primary antibody incubations were performed in blocking buffer overnight at 4 °C. Antibody concentrations used were: Anti-myc (Sigma), 1:2000 dilution; anti-FLAG (Sigma), 1:2000 dilution; anti-β-catenin (Cell Signaling Technologies), 1:2000; anti-Calnexin (AbCam), 1:3000 dilution; rabbit anti-LRRK2 (Epitomics ‘MJFF2’), 1:1000; and mouse anti-LRRK2 (NeuroMab ‘N138/6’), 1:1000. The following morning, membranes were washed three times in TBS-T, prior to incubation in HRP-conjugated secondary antibody (Santa Cruz) at 1:2000 in blocking buffer for 1 h at room temperature. After an additional three washes, staining was visualized using SuperSignal West Pico Chemiluminescent Substrate or SuperSignal West Femto Chemiluminescent Substrate (both Pierce) and a Syngene GeneGnome Imaging system. Images were quantified using GeneQuant software.

### Luciferase assays

Luciferase assays were performed in a 6-well plate format, using a Promega Dual Luciferase Assay Reporter kit and Turner Instruments 20/20 luminometer, as described previously [16].

### Immunofluorescence and confocal microscopy

Cells were transfected with 500 ng of the relevant plasmid per coverslip. 24 h post-transfection, cells were treated with 10 μM MG132 for 1 h to allow accumulation of β-catenin. Cells were fixed in 4% (*w/v*) paraformaldehyde and stained with antibodies to myc and FLAG (Sigma) as described previously [14]. Alexa-488 and −546 conjugated secondary antibodies were from Invitrogen. 4′,6-diamidino-2-phenylindole stain (DAPI; Invitrogen) was performed at a 300 nM concentration for 5 min. Confocal microscopy was performed using a Zeiss LSM 710 META. All images were taken with a 63× objective. Fluorescence exited by the 488, 543 and 633 nm laser lines of Argon and Helium/Neon lasers was detected separately using only one laser at the time (multitrack function) and a combination of band pass filters (BP 505–530, BP 560–615), long pass (LP 560) filters and meta function (649–798) dependent on the combination of fluorochromes used.

### Statistical analysis

In all cases data are expressed as mean ± SEM, with values considered statistically significant when *p* ≤ 0.05. Student’s t-tests were performed using Microsoft Excel, other statistical software used is indicated.

#### Bone analysis

One way analysis of variance (ANOVA) was used to determine the effects of genotype (wild-type and *Lrrk2* knockout) on normally distributed trabecular bone data using GraphPad Prism 6 (GraphPad Software, Inc., San Diego, CA). For cortical bone, analysis graphs were developed using the programming language “R”, version 3.1.1 (R Foundation for Statistical Computing, Vienna, Austria; http://www.r-project.org). Data distribution’s normality was determined using the Shapiro-Wilk test and the Kolmogorov-Smirnov goodness of fit test. A single factor ANOVA was used to analyse equality of means between groups. Group means were compared using the Tukey-Kramer method.

#### Luciferase assays

Luciferase data in Figs. 2a and 3a, where single pairwise comparisons were made, were analysed by two-tailed student’s t-test. All other experiments were analysed by one-way analysis of variance using SPSS software. Experiments in Figs. 5d, 6a, b and Additional file 1: Figure S10, comparing treatments to a single control (wild-type LRRK2 or DMSO treatment, respectively), were subjected to post-hoc analysis by two-sided Dunnett’s testing. Bonferroni post-hoc analysis was performed in the remaining luciferase remaining experiments.

#### Western blot

Differences between β-catenin expression in wild-type and LRRK2 knockout mouse brain was assessed by two-tailed t-testing. Differences between the levels of transcriptionally active ‘free’ β-catenin in wild-type, *Lrrk2* heterozygous and *Lrrk2* knockout mouse brains was determined by two-way ANOVA using SPSS software for the effect of experiment, genotype and the interaction between experiment and genotype, followed by Bonferroni post-hoc testing for the effect of genotype.

## Results

### Adult *Lrrk2* knockout mice exhibit increased predicted bone strength

Greatly increased bone mineral density has been reported in the tibiae, femurs and spine of *Lrrk1* deficient mice, although the mechanism underlying this osteopetrotic phenotype remains unknown [55]. Our previous data demonstrated that siRNA-mediated knockdown of *LRRK1* or *LRRK2* elicits increased Wnt signalling in HEK293 cells, and it is well established that elevated canonical Wnt signalling causes increased bone mass. In light of these observations we hypothesized that *Lrrk2* knockout animals might also display a bone phenotype consistent with elevated canonical Wnt signalling. Thus micro-computed tomography was used to perform a detailed analysis of bone morphology in tibiae from aged female *Lrrk2* knockout mice.

**Fig. 1:**
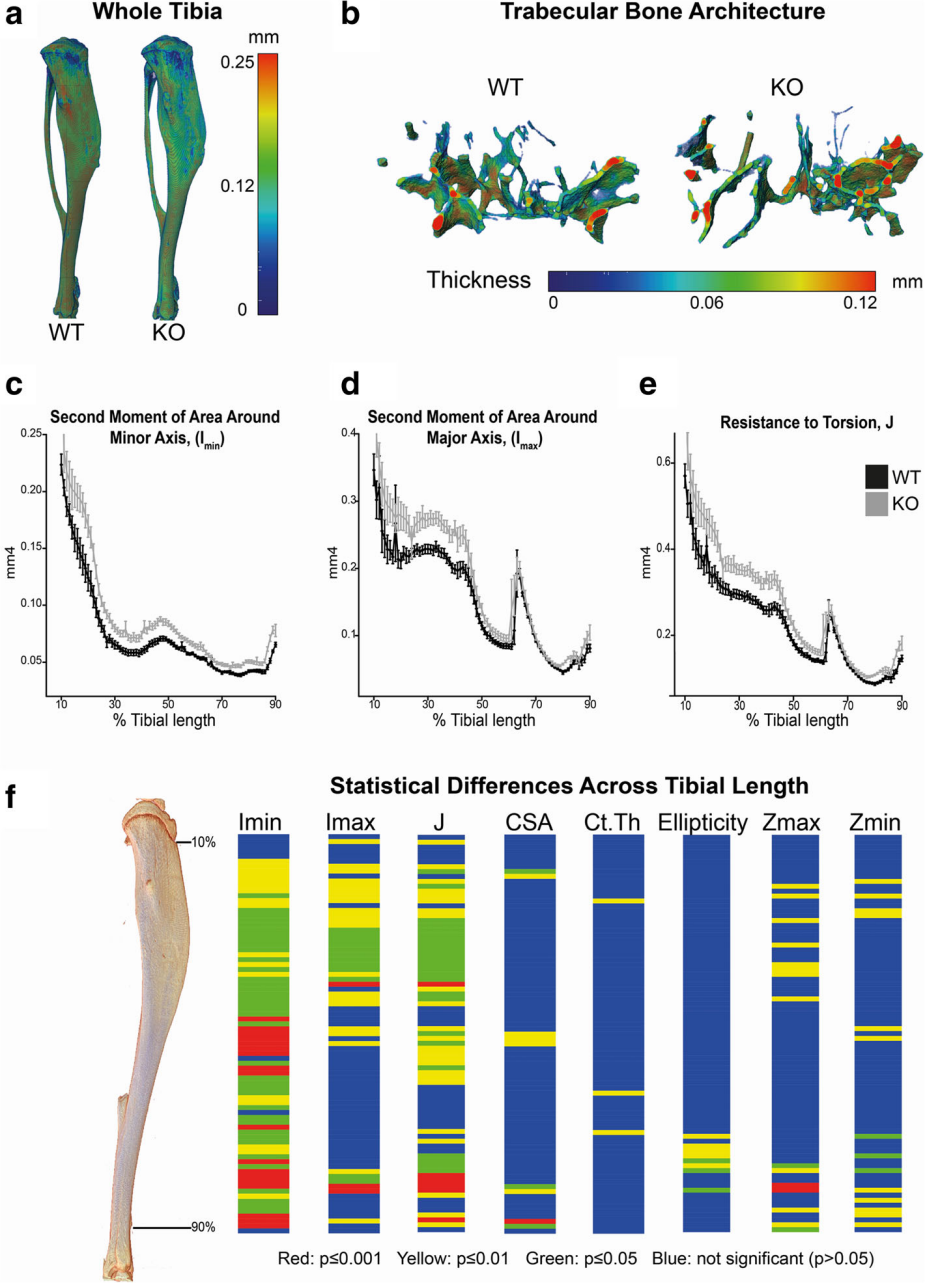
*Lrrk2* knockout mice display increased tibial cortical bone strength. Tibiae from 7 female *Lrrk2* knockout (KO) and 7 age and sex-matched wild-type (WT) mice displayed no differences in **a** gross morphology or **b** trabecular bone architecture. However, micro-computed tomographic analysis of tibial cortical bone revealed **c** increased second moment of area around the minor axis, I_min_; **d** increased second moment of area around major axis, I_max_; and **e** increased predicted resistance to torsion, J. **f** Shows a graphical heatmap displaying statistical differences along the tibia between wild-type and knockout mice, using 1-way ANOVA followed by Tukey-Kramer post-hoc analysis (blue = n/s, yellow, *p* < 0.05, green *p* < 0.01, red, *p* < 0.001). Note that in addition to the parameters I_min_, I_max_ and J, other bone parameters were studied, all of which showed differences (see main text for explanation and Additional file 3: Figure S2 and Additional file 4: Figure S3 for raw data). These altered parameters indicate increased bone strength in *Lrrk2* null animals

We first studied tibial trabecular bone and found no differences in tibial length, trabecular tissue volume (TV), trabecular bone volume (BV), BV/TV, trabecular number and thickness (Fig. 1a-b; data not shown). Cortical bone was next analysed along the entire tibial length, except for the most proximal and distal 10%, as these regions include significant trabecular content. Statistically significant differences between the cortical bones of *Lrrk2* knockout and wild-type mice were observed for multiple parameters (summarized in Fig. 1f). Consistent with a predicted increase in bone strength, the second moment of area around the shortest cross-sectional axis, I_min_, was significantly greater in knockout animals across almost the entire cortical length (Fig. 1c). Increased second moment of area around the longest cross-sectional axis, I_max_, was also observed across most of the bone length (Fig. 1d). Correspondingly, cross sectional area (CSA) was also higher (Additional file 2: Figure S1A), although this only reached statistical significance in a few regions. We observed a differential effect of *Lrrk2* deficiency on the cross sectional thickness (Ct.Th) parameter, with lower values obtained at a proximal region, but significantly higher values at two distal regions (Additional file 2: Figure S1B). *Lrrk2* deficiency also elicited a subtle but statistically significant alteration in tibial shape, as evident by changes in ellipticity of *Lrrk2* knockout tibia in distal regions (Additional file 2: Figure S1C). Notably, predicted resistance to torsion (J) was significantly increased almost across the entire length of *Lrrk2* deficient tibiae (Fig. 1e). Finally, predicted resistance to fracture, as indicated by section modulus Zmax and Zmin (Additional file 3: Figure S2A and B), was also higher in *Lrrk2* deficient mice.

Together these data demonstrate that *Lrrk2* deficiency leads to significant increases in tibial cortical mass and architecture, and consequently improved predicted indices of resistance to torsion and fracture in aged mice. These observations are entirely consistent with the phenotypic consequence of elevated canonical Wnt signalling on bone mass and architecture reported previously [30, 31, 40, 41].

### *Lrrk2* knockout fibroblasts display enhanced β-catenin activation

**Fig. 2:**
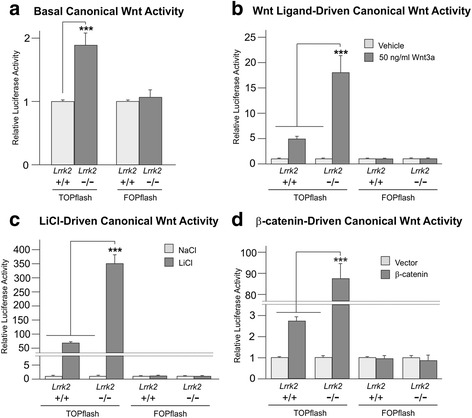
*Lrrk2* knockout cells display increased canonical Wnt activity. **a**-**d** Wild-type and *Lrrk2* knockout cells were co-transfected with TOPflash or FOPflash and TK-renilla. **a** In the absence of canonical Wnt pathway activation, TOPflash activity 24-h post-transfection was significantly higher in *Lrrk2* knockout cells than wild-type controls (*n* = 36; T-test, *p* < 0.001). No difference was detected in FOPflash values (*n* = 30). **b** Overnight transfection followed by 6-h treatment with 50 ng/ml recombinant Wnt3a elicited a further increase in TOPflash activity in *Lrrk2* knockout cells relative to wild-type (1-way ANOVA: *n* = 9; F = 19.143, *p* < 0.001; Bonferroni post-hoc analysis: *p* < 0.001 versus all other conditions). No significant changes in FOPflash values were detected (*n* = 6, F = 0.40, *p* = 0.989). **c** Overnight transfection followed by incubation for 6 h in the presence of 30 mM NaCl or LiCl. 1-way ANOVA (*n* = 9; F = 93.414, *p* < 0.001) followed by Bonferroni post-hoc analysis revealed increased LiCl-driven TOPflash activity in *Lrrk2* knockout cells (*p* < 0.001 relative to all other conditions). No significant changes in FOPflash values were detected (*n* = 6; F = 2.391, *p* = 0.1). **d** Co-transfection with FLAG-β-catenin or empty vector revealed a marked activation of TOPflash by β-catenin in *Lrrk2* knockout cells relative to wild-type cells (*n* = 9; 1-way ANOVA (F = 128.490, *p* < 0.001; Bonferroni post-hoc analysis, *p* < 0.001 versus all other conditions). No significant changes in FOPflash values were detected (*n* = 9, F = 0.165, *p* = 0.919)

**Fig. 3:**
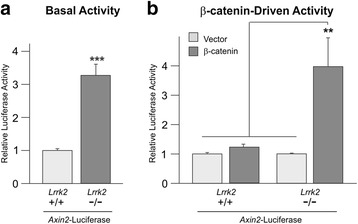
*Lrrk2* knockout cells display increased *Axin2* promoter activity. Wild-type and *Lrrk2* MEFs were co-transfected with a reporter plasmid containing the murine *Axin2* promoter upstream of luciferase and TK-renilla. The relative luciferase activity of resultant cell lysates was measured 24 h post-transfection. **a** In the absence of canonical Wnt pathway activation, *Axin2* promoter activity was significantly higher in *Lrrk2* knock-out cells than wild-type controls (*n* = 18; T-test, *p* < 0.001). **b** Co-transfection with FLAG-β-catenin or empty vector revealed a marked activation of the *Axin2* reporter by β-catenin in *Lrrk2* knockout cells (*n* = 9; 1-way ANOVA (F = 8.037, *p* < 0.001) followed by Bonferroni post-hoc analysis, *p* < 0.01 versus all other conditions)

To investigate the effect of LRRK2 loss-of-function on Wnt signalling more fully, fibroblasts derived from *Lrrk2* knockout and wild-type mice [51] were studied using TOPflash reporter assays [56], which measure β-catenin-driven transcription to infer levels of transcriptionally active β-catenin (i.e. levels of canonical Wnt activity). In agreement with data from siRNA-mediated knockdown of LRRK2 [16], *Lrrk2* knockout cells displayed a small (~1.8-fold) but significant increase in basal TOPflash activity compared to wild-type cells (*p* < 0.001; Fig. 2a). By contrast, the control FOPflash reporter behaved almost identically in both cell types (Fig. 2a). Importantly, the activity of the *Axin2* promoter – a well described canonical Wnt target – was also elevated in *Lrrk2* knockout cells (Fig. 3a).

TOPflash assays were next used to investigate the effect of loss of Lrrk2 on Wnt signalling following treatment with a number of stimuli, namely recombinant Wnt3a (Fig. 2b), LiCl (Fig. 2c), or co-transfection of luciferase reporters with plasmids encoding FLAG-β-catenin (Fig. 2d), FLAG-DVL1 (Additional file 4: Figure S3A), LRP6-HA and EGFP-FZD5 (Additional file 4: Figure S3B), or HA-CK1ε (Additional file 4: Figure S3C). Even correcting for the underlying difference in basal β-catenin activity, each of these treatments elicited a markedly greater induction of the TOPflash reporter plasmid in *Lrrk2* knockout cells. Differences between values obtained in *LRRK2* null cells following pathway activation and all other TOPflash values were highly significant in all cases (Fig. 2b-d and Additional file 4: Figure S3A-B, *p* < 0.001; Additional file 4: Figure S3C, *p* < 0.01). Indeed, in the case of FLAG-β-catenin transfection, the activation of canonical Wnt activity was over 30 times higher than in wild-type cells (wild-type ~2.7x, knockout ~87x; Fig. 2d). By contrast, values from FOPflash were unaffected by all treatments except co-transfection of LRP6-HA and EGFP-FZD5 (Additional file 4: Figure S3B), which mildly repressed the reporter in knockout cells. This indicates that the true activation of Wnt signalling by this treatment may be higher than measured with TOPflash. In agreement with these data, the *Axin2* promoter was also considerably more sensitive to FLAG-β-catenin overexpression in knockout cells (Fig. 3b). Thus in agreement with siRNA-mediated knock-down [16] and the bone phenotype present in *Lrrk2* knockout animals (Fig. 1 , Additional file 2: Figure S1 and Additional file 3: Figure S2), loss of Lrrk2 in fibroblasts leads to an enhancement of both basal and stimulated canonical Wnt activity.

### LRRK2 interacts with and represses β-catenin

The interaction between LRRK2 and β-catenin was next investigated using confocal microscopy to examine the co-localisation of FLAG-β-catenin and mycLRRK2 in HEK293 cells. Owing to the short half-life of β-catenin [57], cells were treated with the proteosome inhibitor MG132 for 1 h prior to fixation. As previously reported, myc-staining revealed the presence of LRRK2 throughout the cytoplasm, but not in the nucleus (Additional file 5: Figure S4A). By contrast, FLAG-staining showed β-catenin to display a prominent nuclear localisation, likely a consequence of the accumulation of this protein caused by proteosomal inhibition. Importantly however, β-catenin was also observed throughout the cytosol, and displayed a strong co-localisation with LRRK2 in this compartment. Consistent images could be achieved using antibodies directed to LRRK2 (data not shown) and β-catenin (Additional file 5: Figure S4B). Interestingly, co-localisation was most pronounced in punctate structures that were not readily detectible when either protein was transfected individually. The nature of these structures is under investigation, but it is likely they are relevant to Wnt signalling, since we also found LRRK2 to display considerable co-localisation with the β-catenin destruction complex components AXIN1 (Additional file 6: Figure S5A) and GSK3β (Additional file 6: Figure S5B). Indeed, similarly to established β-catenin destruction complex proteins, LRRK2 displayed a striking recruitment into polymeric AXIN1 structures [58, 59]. These observations are consistent with our previous finding that Lrrk2 interacts with AXIN1, β-catenin and GSK-3 β endogenously [16], although we acknowledge that co-localisation data from over-expressed proteins should always be treated with caution.

To verify that co-localisation with β-catenin is representative of physical interaction rather than close spatial proximity, co-immunoprecipitations were performed. These assays confirmed that exogenous LRRK2 and β-catenin exist in complex in HEK293 cells (Additional file 7: Figure S6A). Importantly, co-immunoprecipitation was performed in the absence of proteosome inhibition, indicating that the co-localisation observed by confocal microscopy was not an artefact of MG132 treatment. Thus, in agreement with co-immunoprecipitation experiments that were performed in the reciprocal direction on endogenous protein from mouse brain extracts [16], LRRK2 and β-catenin appear to interact strongly in cells.

Since loss of Lrrk2 in vitro had a marked stimulatory effect on multiple measures of Wnt signalling (Figs. 2, 3, and Additional file 4: Figure S3), our data suggested LRRK2 binding to β-catenin protein was exerting an inhibitory influence on this Wnt signalling effector. This idea was investigated further by performing a reciprocal experiment to that shown in Fig. 2d, examining the effect of LRRK2 over-expression on β-catenin-driven TOPflash activity. Consistent with a high turnover of β-catenin in HEK293 cells, exogenous β-catenin elicited a small (~2.5x) activation of TOPflash activity (Additional file 7: Figure S6B). The extent of this activation is similar to the ~2.7-fold activation observed in wild-type fibroblasts (Fig. 2d). In agreement with previous data [16], over-expression of LRRK2 alone had no significant effect on this system. However, consistent with the idea that LRRK2 inhibits β-catenin, co-transfection of LRRK2 with β-catenin produced a marked reduction in the capacity of β-catenin to activate the TOPflash reporter (Additional file 7: Figure S6B).

### Increased β-catenin in the *Lrrk2* knockout brain

Our data suggested LRRK2 binds and represses the transcriptional activity of β-catenin, with loss of LRRK2 relieving this repression, thereby causing elevated canonical Wnt signalling. This idea is consistent with the role for LRRK2 in the β-catenin destruction complex that we postulated previously [16]. Since the best-described role for LRRK2 is in Parkinson’s disease, we sought to corroborate this idea in mouse brain.

**Fig. 4:**
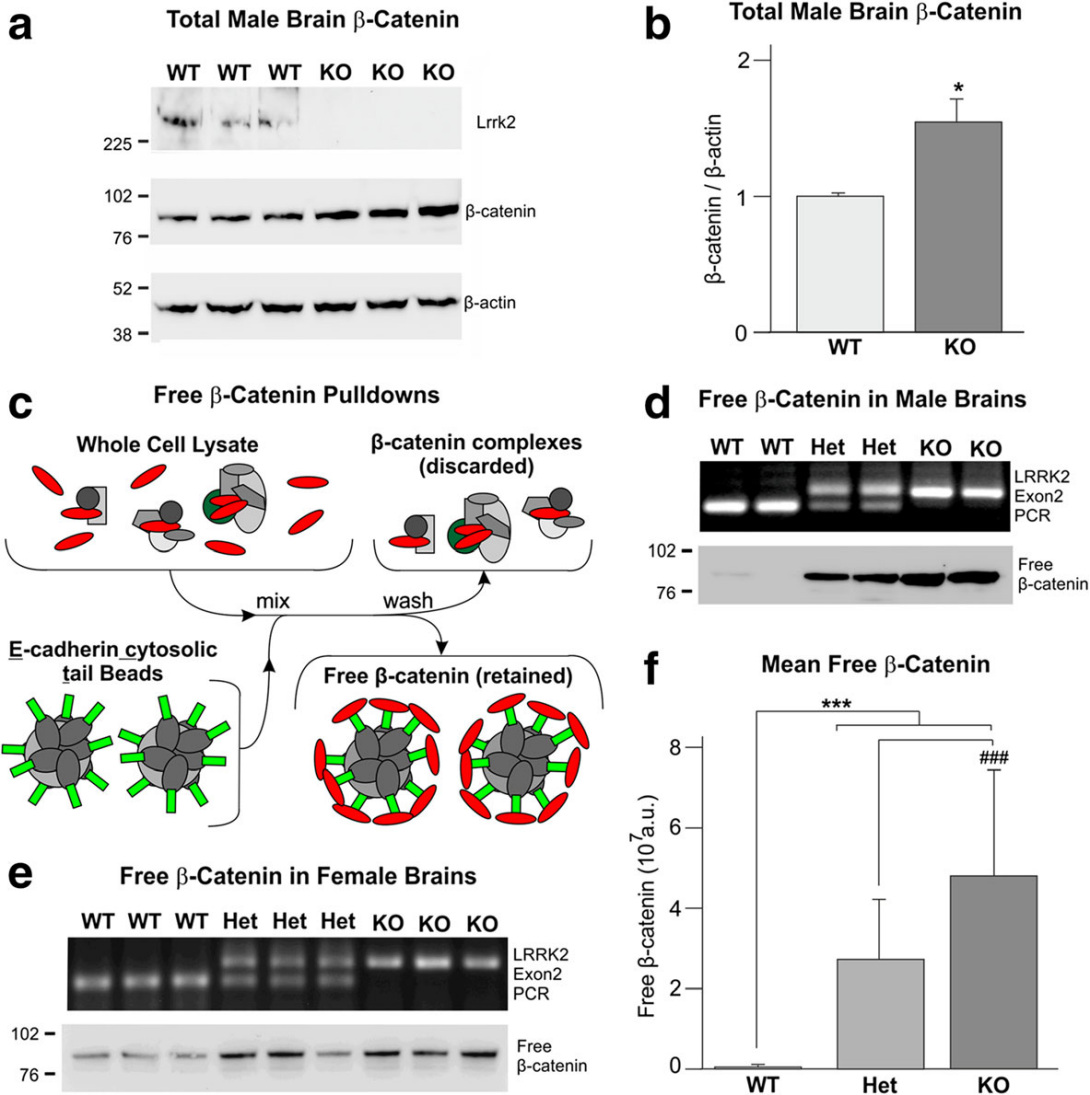
*Lrrk2* knockout mice display elevated β-catenin levels in the brain. Whole brain lysates from aged-matched *Lrrk2* knockout (KO) and wild-type (WT) male mice were resolved by SDS-PAGE and blotted, as indicated, for Lrrk2, β-catenin and β-actin as a loading control. **a** Shows representative images for 3 wild-type and 3 knockout mice. **b** Mean β-catenin/β-actin ratios for ten wild-type and 11 knockout mice, ± the standard error of the mean. These calculations revealed significantly increased β-catenin levels in *Lrrk2* knockout mouse brains (*t*-test, *p* = 0.015). **c** Graphical illustration of ECT assays. Immobilised E-cadherin cytosolic tail protein (*green*) bound to beads can be used to affinity purify free β-catenin (*red*) from complexed β-catenin in lysates. **d**, **e** ECT assays of lysates from male and female *Lrrk2* knockout and heterozygous brains reveal increased free β-catenin in knockout brains with intermediate levels in heterozygotes. **f** Analysis of all ECT data by 2-way ANOVA followed by Bonferroni post-hoc testing reveals significant effects of *Lrrk2* deficiency on free β-catenin levels in the brain

Western blotting of whole brain lysates from 22 to 26 week old male *Lrrk2* null mice revealed a small increase in total β-catenin levels relative to wild-type controls (Fig. 4a, b, and Additional file 8: Figure S7). Although this data is in agreement with LRRK2 repressing β-catenin, it is well established that free β-catenin – the transcriptionally active form of β-catenin that is the output of canonical Wnt signalling – only represents a fraction of total β-catenin. As such, altered total β-catenin may be caused by changes in other pools of this protein. In addition, should canonical Wnt signalling be altered, the presence of other pools of β-catenin diluting the transcriptionally active fraction of β-catenin mean that changes in total β-catenin may not reflect the true extent of altered Wnt signalling activity. Perhaps in agreement with this, although basal Wnt signalling is elevated in Lrrk2 KO MEFS (Figs. 2a and 3a), we failed to see reproducible changes in total β-catenin levels in these cells (data not shown). To address these issues we adapted the E-cadherin cytosolic tail (ECT) assays developed by Luckert and colleagues for use in brain extracts [46]. These assays take advantage of the fact that ECT binds free β-catenin with high affinity, but is unable to bind β-catenin in complex with other proteins. As illustrated in Fig. 4c, recombinant ECT expressed as a GST fusion protein and coupled to glutathione-Sepharose beads can be used as an effective tool to purify free β-catenin from cell lysates. Importantly, data from ECT assays correlate well with parallel TOPflash assays [46].

ECT assays performed on brain extracts from a set of male *Lrrk2* knockout, *Lrrk2* heterozygous and wild-type mice revealed a marked increase in the levels of free β-catenin with loss of Lrrk2 (Fig. 4d, Additional file 9: Figure S8A). These data also suggested a gene dosage effect, with free β-catenin levels higher in knockout than heterozygous brains. We sought to replicate these observation using material from aged female animals consistent with the cohort used in bone morphology analysis (Fig. 1). The differences between groups are less than observed in younger male animals, and consistently we were unable to detect differences in total β-catenin levels (Additional file 9: Figure S8B). Nonetheless, aged *Lrrk2* deficient female brains still showed increased free β-catenin, with heterozygotes displaying an intermediate level (Fig. 4e, Additional file 9: Figure S8C). Analysis of the combined data using 2-way ANOVA confirmed the significant effect of genotype on free β-catenin (F = 384.945, *p* < 0.001; Fig. 4f), as well as significant effects of experiment and the interaction between genotype and experiment on this variable. Importantly, Bonferroni post-hoc testing revealed that the differences between all three genotypes are highly significant (*p* < 0.001 for all pairwise comparisons). Thus, in addition to causing an increase in total β-catenin in the brains of younger male *Lrrk2* knockout mice, loss of Lrrk2 causes a dose-dependent elevation of free β-catenin in the brains of both younger males and aged females, albeit to different extents. Taken together, these data provide strong evidence for elevated canonical Wnt signalling in the brains of *Lrrk2* deficient animals.

### LRRK2 kinase inhibition weakens basal canonical Wnt signalling

We previously reported that the LRRK2 kinase inhibitor LRRK2-in-1 [52] represses TOPflash activity driven by DVL1 over-expression in SH-SY5Y cells, when applied at 1 μM [16]. However, this compound is known to inhibit other kinases, suggesting that repression of Wnt signalling may not be specific to LRRK2. This was a major concern since canonical Wnt signalling is regulated by a large number of kinases [60]. Thus, the experiment was repeated over a concentration gradient, to look for effects at concentrations closer to the reported IC50 for the compound, and in a second cell line, HEK293 cells. In addition, two unrelated LRRK2 kinase inhibitors, TAE684 [53] and CZC25146 [54], both of which have also been reported to display off-target effects, were studied in parallel.

**Fig. 5:**
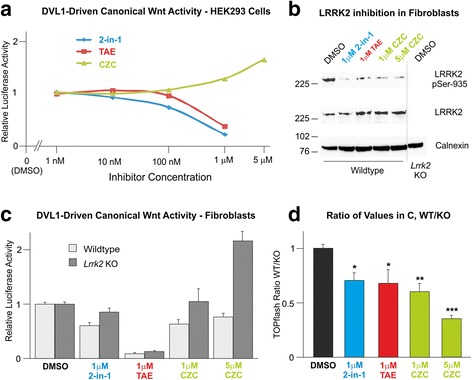
Effect of LRRK2 kinase inhibition on canonical Wnt signalling. **a** HEK293 cells transfected with TOPflash and FLAG-DVL1 were treated with DMSO or LRRK2-in-1, TAE684, or CZC-25146 for 20 h at the indicated concentrations. Resultant luciferase values are expressed relative to DMSO controls. **b**-**d** The effect of kinase inhibion with these compounds was further analysed by comparing effects in wild-type and *Lrrk2* knockout (KO) cells. **b** At concentrations used LRRK2 phosphorylation at serine-935 is reduced. **c** Using conditions identical to panel A, the three compounds also display differing effects in wild-type and *Lrrk2* knockout cells. **d** The values produced for each inhibitor in panel C were expressed as a ratio of wild-type cells over knockout cells, indicating that LRRK2 inhibition weakens DVL1-driven canonical Wnt signalling. 1-way ANOVA reveals a significant effect of treatment (*n* = 12, F = 8.628, *p* < 0.001), with 2-sided Dunnett’s post-hoc analysis indicating significant differences between all treatments relative to control (LRRK2-in-1, *p* < 0.05; TAE684, *p* < 0.05; 1 μM CZC-25146, *p* < 0.01; 5 μM CZC-25146, *p* < 0.001)

In agreement with previous data, 1 μM LRRK2-in-1 produced a robust inhibition of DVL1-driven TOPflash activity (~12% of DMSO control; Fig. 5a), with a mild effect apparent at 100nM (~68% of DMSO). This is consistent with the reported in vitro IC50 for this compound of 13 nM [52]. Strong TOPflash inhibition was also seen with 1 μM TAE684 (~29% of DMSO). Surprisingly however, CZC25146 had the opposite effect, inducing a small activation of TOPflash at this concentration (~128% of DMSO). To corroborate this observation, the CZC25146 concentration gradient was extended to 5 μM for the final repeat. Under these conditions it was clear that in contrast to the two other LRRK2 kinase inhibitors, CZC25146 stimulates the canonical Wnt pathway.

Although two of the three inhibitors gave data consistent with our previous observation, the contrasting effects of CZC25146 was a great concern, indicating that off-target kinase inhibition was affecting our data. We thus decided to compare the effects of the compounds by performing parallel TOPflash assays in *Lrrk2* knockout and wild-type fibroblasts. Since only wild-type cells express Lrrk2, values obtained from these cells are the product of both on- and off-target kinase inhibition, whilst values from knockout cells can only be the product of off-targeting. Our objective was to express the values recorded in both cell lines as a ratio from which the specific effect of LRRK2 kinase inhibition can be inferred. Following our observations made in Fig. 5a, all three compounds were applied at 1 μM, with CZC25146 also studied at 5 μM. Under these conditions Lrrk2 kinase activity was efficiently suppressed, as evidenced by decreased phosphorylation in wild-type cells of Lrrk2 at serine-935, which is known to be dependent on LRRK2 kinase activity (Fig. 5b and Additional file 10: Figure S9; [52]).

TOPflash assays performed in wildtype and *Lrrk2* knockout cell lines allowed two observations to be made. Firstly, as evidenced by the inhibitory and stimulatory effects of TAE264 and CZC25146 respectively in *Lrrk2* null cells, both compounds clearly exhibit off-target effects in these assays (Fig. 5c). Secondly however, comparison of the TOPflash values obtained in both cell lines as a ratio to account for non-specific effects yields consistent results for all three compounds (Fig. 5d). Remarkably, at 1 μM the three LRRK2 kinase inhibitors elicited comparable and statistically significant decreases in TOPflash activity (2-in-1 ~ 71%, TAE684 ~ 68%, and CZC25146 ~ 60% relative to DMSO). Treatment with 5 μM CZC25146 yielded an even greater inhibition (~35% of DMSO). In conclusion therefore, pharmacological inhibitors of LRRK2 produce disparate effects on DVL1-driven canonical Wnt signalling that are at least in part caused by non-specific kinase inhibition. As such, these data must be treated with caution. However, our data suggest strongly that the inhibition of LRRK2 kinase activity *per se* represses activity of this signalling cascade.

We have previously reported that LRRK2 mutants containing kinase-dead and GTP-non-binding amino-acid substitutions reduced the capacity of over-expressed LRRK2 to enhance Dvl1-driven Wnt signalling [16]. These assays have produced consistent data, but are clearly not reflective of physiological Wnt signalling. To address this issue we chose to investigate the effect of over-expressing LRRK2 mutants on TOPflash activity under tonic conditions. Such assays have proven troublesome in other cell types due to the poor signal-to-noise ratios achieved in the absence of pathway stimulation (data not shown). However, since *Lrrk2* knockout fibroblasts have elevated basal TOPflash activity, we hypothesized that these cells might be a suitable model for these experiments. Furthermore, assaying the effects of LRRK2 mutations in knockout cells has the additional advantage of removing competition from endogenous Lrrk2 protein.

The effect of artificial kinase-dead mutations in LRRK2 was thus tested in *Lrrk2* knockout cells. In agreement with data from DVL1-driven TOPlash assays, kinase inactivation was found to inhibit basal Wnt activity by at least 50% relative to wild-type LRRK2 (Additional file 1: Figure S10). Two further artificial LRRK2 mutants were assayed: K1347A and T1348N. Both of these variants abrogate the binding of the LRRK2 RocCOR domain to guanyl nucleotides. We have previously reported that K1347A inhibits the effect of LRRK2 over-expression on DVL1-driven TOPflash activity similarly to kinase-dead mutations [16]. To our surprise, neither K1347A nor T1348N produced any statistically significant effects on basal TOPflash activity (Additional file 1: Figure S10).

### PD-causing *LRRK2* mutations weaken basal Wnt signalling

**Fig. 6:**
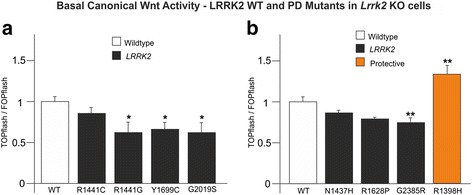
Effect of *LRRK2* mutations on basal Wnt signalling. *Lrrk2* knockout (KO) cells were transfected with TOPflash or FOPflash plus wild-type LRRK2 or the indicated LRRK2 mutant. **a** 1-way ANOVA (*n* = 9-15, F = 3.296, *p* < 0.05) followed by 2-sided Dunnett’s post-hoc analysis indicate that pathogenic *LRRK2* mutations weaken canonical Wnt signalling relative to wild-type LRRK2 (RG, *p* < 0.05; YC, *p* < 0.05; GS, *p* < 0.05). RC is decreased relative to wild-type but does not reach significance. **b** 1-way ANOVA (*n* = 9, F = 13.388, *p* < 0.001) followed by 2-sided Dunnett’s post-hoc analysis indicate the pathogenic *LRRK2* mutation GR weakens basal canonical Wnt signalling relative to wild-type LRRK2 (*p* < 0.01), whilst the protective RH mutation enhances canonical Wnt activity (*p* < 0.01). Note that the pathogenic mutations NH and RP decreased relative to wild-type but neither reaches significance. Mutations used: RC = R1441C, RG = R1441G, YC = Y1699C, GS = G2019S, NH = N1437H, RP = R1628P, GR = G2385R; RH = R1398H

Finally, we sought to use *Lrrk2* knockout fibroblasts to determine the effect of pathogenic *LRRK2* mutations on basal canonical Wnt signalling. We firstly tested R1441C, Y1699C and G2019S, which we have previously reported to weaken the effect of LRRK2 over-expression on DVL1-driven TOPflash activity [16]. The pathogenic R1441G mutant was assayed in parallel. Remarkably, all four PD-causing mutations weakened basal canonical Wnt signalling (Fig. 6a), although the effect of R1441C did not reach statistical significance. In light of this result, three further PD-associated variants, N1437H, R1628P and G2385R, were examined in this assay, alongside a LRRK2 construct containing the protective R1398H mutation. Each of these pathogenic variants qualitatively weakened Wnt signalling, although only the effect of G2385R reached significance (Fig. 6b). Remarkably however, R1398H produced the opposite result: a statistically significant *increase* in TOPflash activity. Importantly, the data from the G2385R and R1398H mutants corroborate results from our DVL1-driven TOPflash model [18]. Taken together, these data provide strong evidence that PD-causing mutations throughout *LRRK2* weaken the activity of the canonical Wnt pathway under tonic conditions. In stark contrast, the PD-protective R1398H variant increases the strength of this pathway.

## Discussion

Herein, we present evidence that loss of LRRK2 leads to elevated canonical Wnt signalling in vivo. We report for the first time that *Lrrk2* knockout mice exhibit elevated levels of transcriptionally active free β-catenin in the brain (Fig. 4), and show a corresponding increase in predicted bone strength in aged mice (Fig. 1). Accordingly, *Lrrk2* knockout fibroblasts display elevated canonical Wnt activity – both basally and following pathway stimulation (Fig. 2) – as well as increased activity of the *Axin2* promoter, a canonical Wnt target (Fig. 3). Providing a mechanistic rationale for these observations, LRRK2 binds β-catenin and represses the transcriptional activity of this protein (Additional file 5: Figure S4, Additional file 6: Figure S5 and Additional file 7: Figure S6). Taken together, these data are consistent with a role for LRRK2 as a component of the β-catenin destruction complex. Using fibroblasts derived from knockout mice, we also performed a thorough characterisation of the effects of LRRK2 kinase inhibition on canonical Wnt signalling, finding strong evidence that such compounds suppress the pathway (Fig. 5). Finally, we show that under basal conditions, PD causing *LRRK2* mutations repress canonical Wnt activity, whilst the protective R1398H mutation has the opposite effect (Fig. 6). These last observations suggest that in the context of canonical Wnt signalling, pathological *LRRK2* mutations are gain-of-function, enhancing the repression of β-catenin mediated by LRRK2.

Our data add to the growing body of work implicating deregulated canonical Wnt activity in neurodegeneration in general and Parkinson’s disease in particular. In addition to LRRK2, a remarkable number of genes linked to PD have been shown to modulate the canonical Wnt pathway, for example VPS35, PINK1, UCHL-1, Parkin, ATP6AP2, and GBA [61–66]. Furthermore, the integral Wnt component GSK3β has been implicated in the modulation of PD risk [67]. Supporting this idea, the differentiation of the dopaminergic neurones of the substantia nigra that are lost in PD appears to be exquisitely sensitive to Wnt signalling [68, 69]. Furthermore, Wnt signalling is vital for adult neurogenesis [70] and for the normal function of mature neurones [71, 72]. Since the neuroprotective capacity of canonical Wnt signalling is also well described [27, 71, 73] and altered Wnt signalling has already been reported in the brains of PD patients and model animals [74–76], modifying Wnt activity is evidently a plausible therapeutic strategy for PD.

In this report we provide the first evidence that *Lrrk2* deficiency alters bone mass and architecture, which is consistent with a mild elevation in canonical Wnt signalling. It is very difficult to speculate which mechanisms and cell types are involved, since the canonical Wnt signalling pathway regulates bone mass through a number of different mechanisms. These include renewal of stem cells [77], stimulation of osteoblast differentiation and proliferation [40], enhancement of osteoblast activity [31, 40], inhibition of osteoblast and osteocyte apoptosis [30], regulation of osteoclastogenesis [36, 39] and modulation of adaptive responses to mechanical strain [41, 78–82]. This last phenomenon is particularly intriguing in the context of a gene involved in Parkinson’s disease, since it creates a mechanism whereby altered bone strength may be manifest with ageing.

We note that our study is not the first to assess bone microarchitecture in *Lrrk2* deficient animals, and at first glance the two reports appear in conflict. Specifically, Xing and colleagues found bones from *Lrrk2* knockout mice to be no different to wild-type – albeit with the important caveat that 8-week old males were used, whereas we used 60–64 week old females [55]. The authors, however, reported that targeted disruption of *Lrrk1* leads to severe osteopetrosis. This age and sex difference is clearly sufficient to account for any discord. However, closer inspection indicates that the two studies are in fact in agreement, since Xing and colleagues only examined trabecular bone [55]. We also found no phenotype in this bone type (Fig. 1a, b), with evidence of increased bone strength only present in cortical bone. Evidently, future studies will need to compare the bone parameters of *Lrrk1* and *Lrrk2* null mice directly, using both cortical and trabecular bone, and in males and females over a range of ages.

It would also be informative to study *Lrrk1/Lrrk2* double knockout animals in parallel, but what bone phenotype might one expect? Intuitively one would assume a more severe phenotype, which is supported by previous studies of canonical Wnt pathway components using double knockout animals. For example, the following double knockout mice – *Lef/Tcf1*, *Lrp5/Lrp6*, *Wnt1/Wnt3a*, *Dvl1/Dvl2*, *Krm1/Krm2 –* all display more pronounced developmental defects than corresponding single knockouts [83–89]. Indeed, *Krm1/Krm2* mice show increased bone density, even though *Krm1* and *Krm2* null animals have normal bone parameters [89]. Such predictions must of course be made with caution, especially since we do not know the mechanisms by which Lrrk1 and Lrrk2 affect bone. Nonetheless we believe there is sufficient evidence to expect a more severe phenotype in *Lrrk1/Lrrk2* animals than seen in either single knockout.

The relative subtlety of the *Lrrk2* bone phenotype notwithstanding, our data raise the question as to why anatomical phenotypes associated with elevated canonical Wnt signalling have not been reported previously. In the case of the most studied organ, the brain, decreased canonical Wnt activity produces well described developmental defects [68, 69], but increased Wnt is not associated with an obvious phenotype. It is therefore unsurprising that *Lrrk2* knockout brains display no apparent defects. Nonetheless, Wnt signalling is well known to promote neurite outgrowth and a number of studies have observed increased neurite length in neurones explanted from *Lrrk2* null animals (reviewed in [90]). Thus, loss of *Lrrk2* may produce subtle developmental differences that are presumably transient and recoverable. In contrast to brain, *Lrrk2* knockout animals do have a widely reported kidney phenotype, as well as defects in liver and lung [91–93]. Intriguingly, elevated canonical Wnt signalling is associated with a remarkable range of kidney diseases, including acute kidney injury, diabetic nephropathy, tubulointerstitial fibrosis and polycystic kidney disease [24], and also causes fibrotic conditions in certain other organs, including both liver and lung [94]. As such, the possibility that increased canonical Wnt activation underlies these phenotypes requires investigation, for example by crossing *Lrrk2* deficient mice with animals with decreased Wnt signalling.

Finally, increased predicted bone strength in *Lrrk2* null mice is particularly interesting to consider in light of the growing links between Parkinson’s disease and osteoporosis. It has long been known that PD patients are at increased risk of bone fractures, although this has traditionally been accounted for by the greater risk of falling due to postural instability, together with less exercise creating more fragile bones directly and indirectly via less exposure to sunlight (leading to lower Vitamin D). However, recent advances suggest osteoporosis and PD are more intimately linked, with rates of co-morbidity far higher than assumed [95]. In fact, Invernizzi and colleagues reported osteoporosis or osteopenia (in which bone density is decreased to a lesser extent than osteoporosis) in as much as 91% of female PD patients and 61% of males [96]. A recent meta-analysis estimated this “osteoporosis or osteopenia in PD” odds ratio as 2.61 compared to healthy controls [97]. As such, it is tempting to speculate that osteoporosis and PD may share some common etiology. We have yet to examine bone parameters in transgenic mice with pathological *Lrrk2* mutations, and we are unaware of any similar studies on human *LRRK2* patients or unaffected carriers. However, our data suggest strongly that these individuals have lowered canonical Wnt activity (Fig. 5; [16, 18]), which in turn would predict decreased bone strength. It would clearly be interesting not only to investigate this hypothesis, but to determine whether altered bone parameters can occur in the absence of (or before) motor symptoms. Given that so many other PD-causing mutations have been reported to repress Wnt signalling [61–63, 65, 66], the links between PD and bone health warrant further investigation. Furthermore, since half of all bone fractures in PD are hip fractures and these injuries carry a bleak 1-year mortality rate of 30% [97], pharmacological treatments that improve neuronal survival and increase bone strength would be particularly welcome.

## Conclusions

Our data indicate that LRRK2 is a repressor of β-catenin and therefore *Lrrk2* deficient mice display elevated canonical Wnt signalling and increased indices of predicted bone strength. Four out of seven pathogenic LRRK2 mutations are associated with statistically significant increases in repression of Wnt activity, whilst a protective mutation has the opposite effect. Thus, this study supports the connection between decreased Wnt signalling and Parkinson’s disease, and suggests that the deregulation of this pathway may not only contribute to neurodegeneration, but may also account for the increased incidence of osteoporosis seen in PD patients.

## Additional files

Additional file 1: Figure S10. Kinase-dead but not GTP-non-binding mutations weaken basal Wnt signalling. *Lrrk2* knockout (KO) cells were transfected with TOPflash or FOPflash plus wild-type LRRK2 or the indicated LRRK2 mutant. 1-way ANOVA (*n* = 12–15, F = 7.449, *p* < 0.001) followed by 2-sided Dunnett’s post-hoc analysis indicate that kinase-dead mutations significantly weaken canonical Wnt signalling relative to wild-type LRRK2 (1994, *p* < 0.05; 2017, *p* < 0.05). Mutations used: kinase-dead: 1994 = D1994A, 2017 = D2017A; guanyl nucleotide-non-binding: KA = K1347A, TN = T1348N. (PDF 46 kb)
Additional file 2: Figure S1. Effects of loss of *Lrrk2* on tibial cortical Cross Sectional Area, Mean Cortical Thickness, and Ellipticity. Images in Figures A, B and C show i) values for cross-sectional area, mean cortical thickness and ellipticity, respectively in female wild-type (WT) and *Lrrk2* knockout (KO) mice; together with ii) the same data expressed as a graphical heat map along the tibial length; and iii) the points at which differences between genotypes for these parameters become statistically significant. (blue = n/s, yellow, *p* < 0.05, green *p* < 0.01, red, *p* < 0.001). (PDF 131 kb)
Additional file 3: Figure S2. Effects of loss of *Lrrk2* on tibial cortical Zmax and Zmin. Images in Figures A and B show i) values for Zmax and Zmin, predicted resistance to fracture along the shortest and longest cross-sectional axes, respectively in female wild-type (WT) and *Lrrk2* knockout (KO) mice; together with ii) the same data expressed as a graphical heat map along the tibial length; and iii) the points at which differences between genotypes for these parameters become statistically significant. (blue = n/s, yellow, *p* < 0.05, green *p* < 0.01, red, *p* < 0.001). (PDF 110 kb)
Additional file 4: Figure S3. Increased canonical Wnt activity in *Lrrk2* knockout cells. A, B) Cells were transfected with TK-renilla and TOPflash or FOPflash in the presence of A) FLAG-DVl1 or C) GFP-FZD5 and HA-LRP6, or appropriate vector controls. A) 1-way ANOVA (*n* = 9; F = 146.199, *p* < 0.001) followed by Bonferroni post-hoc analysis revealed increased DVL1-driven TOPflash activity in *Lrrk2* knockout cells (*p* < 0.001 versus all other conditions). No significant changes in FOPflash values were detected (*n* = 9; F = 2.668, *p* = 0.064). B) 1-way ANOVA (*n* = 9; F = 70.694, *p* < 0.001) followed by Bonferroni post-hoc analysis revealed increased GFP-FZD5/HA-LRP6-driven TOPflash activity in *Lrrk2* knockout cells (*p* < 0.001 versus all other conditions). By contrast, the same treatment elicited a significant *decrease* in FOPflash values (1-way ANOVA: *n* = 6; F = 11.129, *p* = 0.001. Bonferroni post-hoc analysis *p* < 0.001). C) Wild-type and *Lrrk2* MEFs were co-transfected with TK-renilla and TOPflash or FOPflash in the presence of active or inactive (KR) HA-tagged CK1ε. 1-way ANOVA (*n* = 9; F = 7.619, *p* = 0.001) followed by Bonferroni post-hoc analysis revealed increased CK1ε-driven TOPflash activity in *Lrrk2* knockout cells (*p* < 0.01 versus all other conditions). No significant changes in FOPflash values were detected (*n* = 3; F = 1.535, *p* = 0.279). (PDF 90 kb)
Additional file 5: Figure S4. Association of mycLRRK2 and FLAG-β-catenin in HEK293 cells 24 h post transfection, cells expressing myc-tagged LRRK2 and FLAG-tagged β-catenin were treated with MG132 for 1 h to allow β-catenin accumulation. The cells were subsequently fixed and stained with antibodies for A) myc (green) and FLAG (red), or B) myc (green) and β-catenin (red). DAPI staining (blue) was also performed to show cell nuclei. The right-hand panel shows an overlay of all channels – note the considerable co-localisation between LRRK2 and β-catenin in the cytoplasm. (PDF 102 kb)
Additional file 6: Figure S5. Co-localisation between mycLRRK2 and HA-AXIN1, and mycLRRK2 and HA-GSK3β in HEK293 cells. A) Recruitment of mycLRRK2 (green) into polymers formed by HA-AXIN1 (red). Magnified images of a selected region are included. B) shows cytoplasmic co-localisation between mycLRRK2 (green) and HA-GSK3β (red), also with magnified images included. Note that in both experiments, counterstaining with phalloidin (blue) and DAPI (grey) were performed to visualise filamentous actin and chromosomal DNA respectively. The scale bar = 10 μm. (PDF 92 kb)
Additional file 7: Figure S6. LRRK2 interacts with β-catenin and represses β-catenin-driven TOPflash activity. A) HEK293 cells were co-transfected with FLAG-tagged β-catenin (lanes 1 and 4), myc-tagged LRRK2 (lanes 2 and 5), or myc-tagged LRRK2 and FLAG-tagged β-catenin (lanes 3 and 6) for 24 h prior to lysis. Lysates were immunoprecipitated with anti-FLAG antibodies and bound protein was resolved by Western blot (lanes 4–6), with the original lysates run alongside to confirm expression of transfected protein (lanes 1–3). Myc-tagged LRRK2 can clearly be seen in anti-FLAG immunoprecipitates from co-transfected cells (lane 6, upper panel). Full images of all blots are also shown. B) HEK293 cells were transfected with the TOPflash or FOPflash reporter plasmids with the indicated combinations of mycLRRK2, FLAG-β-catenin or appropriate control vectors. After 24 h lysates were taken and luciferase activity measured. 1-way ANOVA of TOPflash values revealed a significant effect of transfection on canonical Wnt activity (*n* = 9, F = 44.893, *p* < 0.001). Post-hoc Bonferroni testing showed significantly increased Wnt signalling caused by FLAG-β-catenin transfection in the presence or absence of mycLRRK2 co-transfection (*p* < 0.001 in both cases). Importantly however, co-transfected mycLRRK2 significantly weakened the TOPflash activation elicited by FLAG-β-catenin (*p* < 0.001). ANOVA of control FOPflash values also revealed significant effects of treatment, however Bonferroni post-hoc analysis found no significant differences in any pair-wise comparison. (PDF 129 kb)
Additional file 8: Figure S7. Entire images of Western Blots shown in Fig. 4a. (PDF 70 kb)
Additional file 9: Figure S8. Entire images of Western Blots shown in Fig. 4D and E and total β-catenin levels in aged female mouse brains A) Full blot from the image shown in Fig. 4d, plus a reprobe of the same membrane for calnexin to show that pulldowns are clean and this protein is only present in cell lysates. B) Western blotting shows no discernible differences between levels of total β-catenin in brains from aged female mice. C) Full blot from the image shown in Fig. 4e. (PDF 102 kb)
Additional file 10: Figure S9. Entire images of Western Blots shown in Fig. 5b. (PDF 80 kb)

## Abbreviations

ANOVA: Analysis of variance
CK1ε: Casein kinase 1 epsilon
COR: C-terminal of Roc
CT: Computed tomography
DVL: Dishevelled
ECT: E-cadherin cytosolic tail
FZD5: Frizzled receptor 5
GSK3β: Glycogen synthase kinase 3 beta
GST: Glutathione S transferase
KO: Knockout
LRP6: Low density lipoprotein receptor related protein 6
LRRK2: Leucine-rich repeat kinase 2
PBS: Phosphate buffered saline
PD: Parkinson’s disease
Roc: Ras of complex proteins
SEM: Standard error of the mean
Wnt: Wingless/Int
WT: Wild-type
