# Supplementary figures and images for: Pathogenic LRRK2 variants are gain-of-function mutations that enhance LRRK2-mediated repression of β-catenin signaling
Source: Mol Neurodegener. 2017 Jan 19;12:9. doi: 10.1186/s13024-017-0153-4 (PMC5248453; doi:10.1186/s13024-017-0153-4)

Supplementary Figure 10

**Basal Canonical Wnt Activity - *Lrrk2* KO cells**

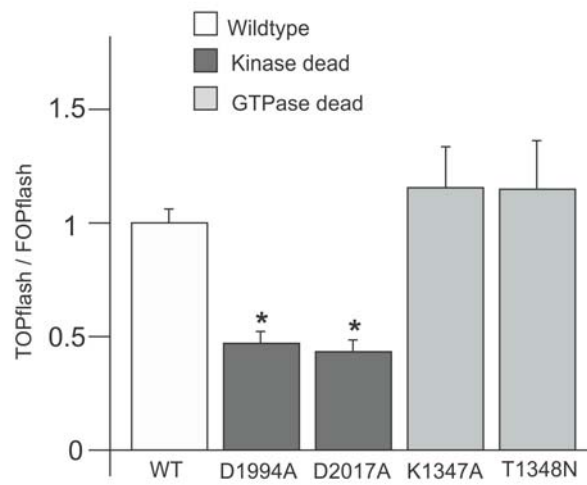

Supplement: Additional file 1: Figure S10. — Kinase-dead but not GTP-non-binding mutations weaken basal Wnt signalling. Lrrk2 knockout (KO) cells were transfected with TOPflash or FOPflash plus wild-type LRRK2 or the indicated LRRK2 mutant. 1-way ANOVA (n = 12–15, F = 7.449, p < 0.001) followed by 2-sided Dunnett’s post-hoc analysis indicate that kinase-dead mutations significantly weaken canonical Wnt signalling relative to wild-type LRRK2 (1994, p < 0.05; 2017, p < 0.05). Mutations used: kinase-dead: 1994 = D1994A, 2017 = D2017A; guanyl nucleotide-non-binding: KA = K1347A, TN = T1348N. (PDF 46 kb) [file 13024_2017_153_MOESM1_ESM.pdf]

Supplementary Figure 1

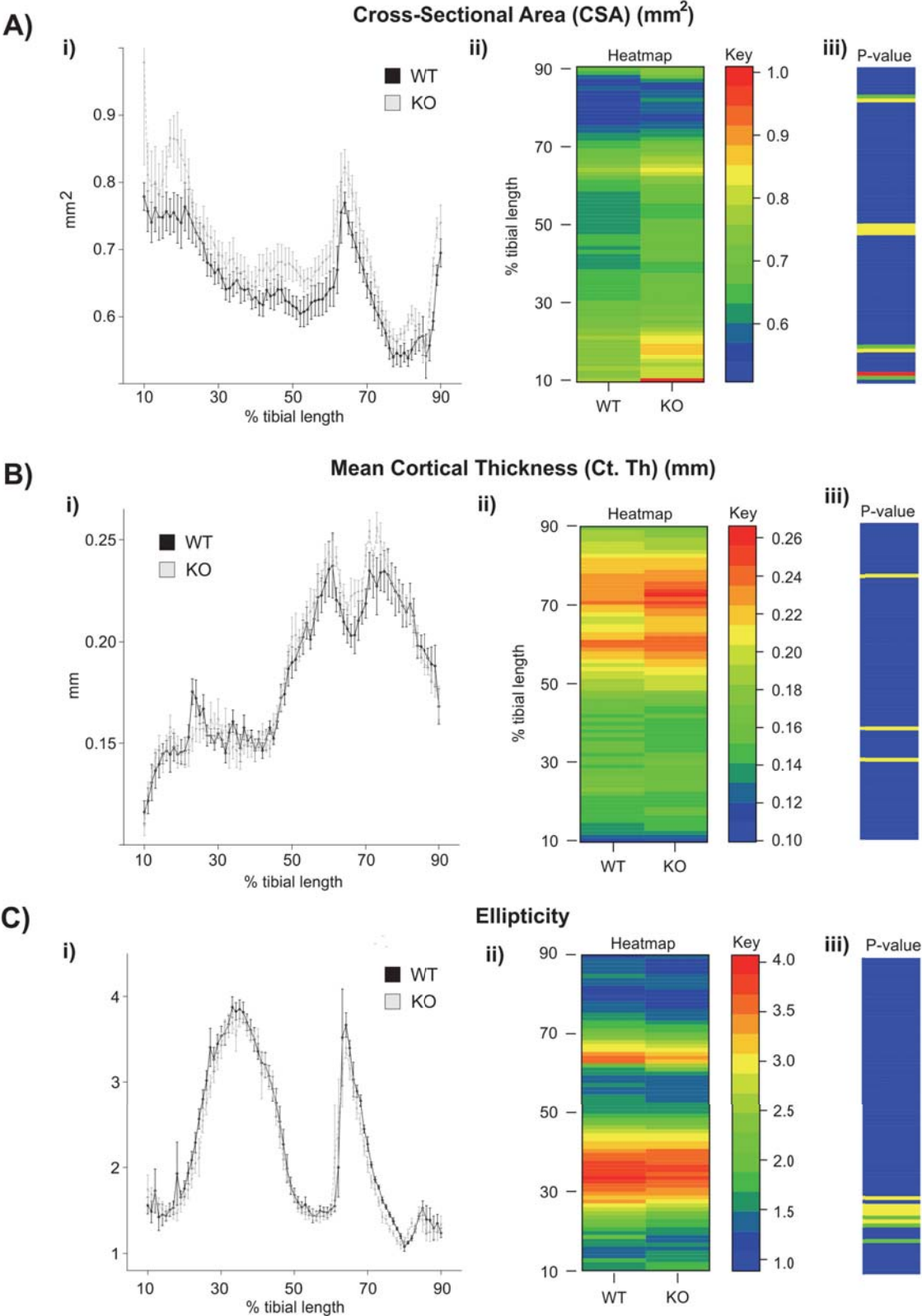

Supplement: Additional file 2: Figure S1. — Effects of loss of Lrrk2 on tibial cortical Cross Sectional Area, Mean Cortical Thickness, and Ellipticity. Images in Figures A, B and C show i) values for cross-sectional area, mean cortical thickness and ellipticity, respectively in female wild-type (WT) and Lrrk2 knockout (KO) mice; together with ii) the same data expressed as a graphical heat map along the tibial length; and iii) the points at which differences between genotypes for these parameters become statistically significant. (blue = n/s, yellow, p < 0.05, green p < 0.01, red, p < 0.001). (PDF 131 kb) [file 13024_2017_153_MOESM2_ESM.pdf]

Supplementary Figure 2

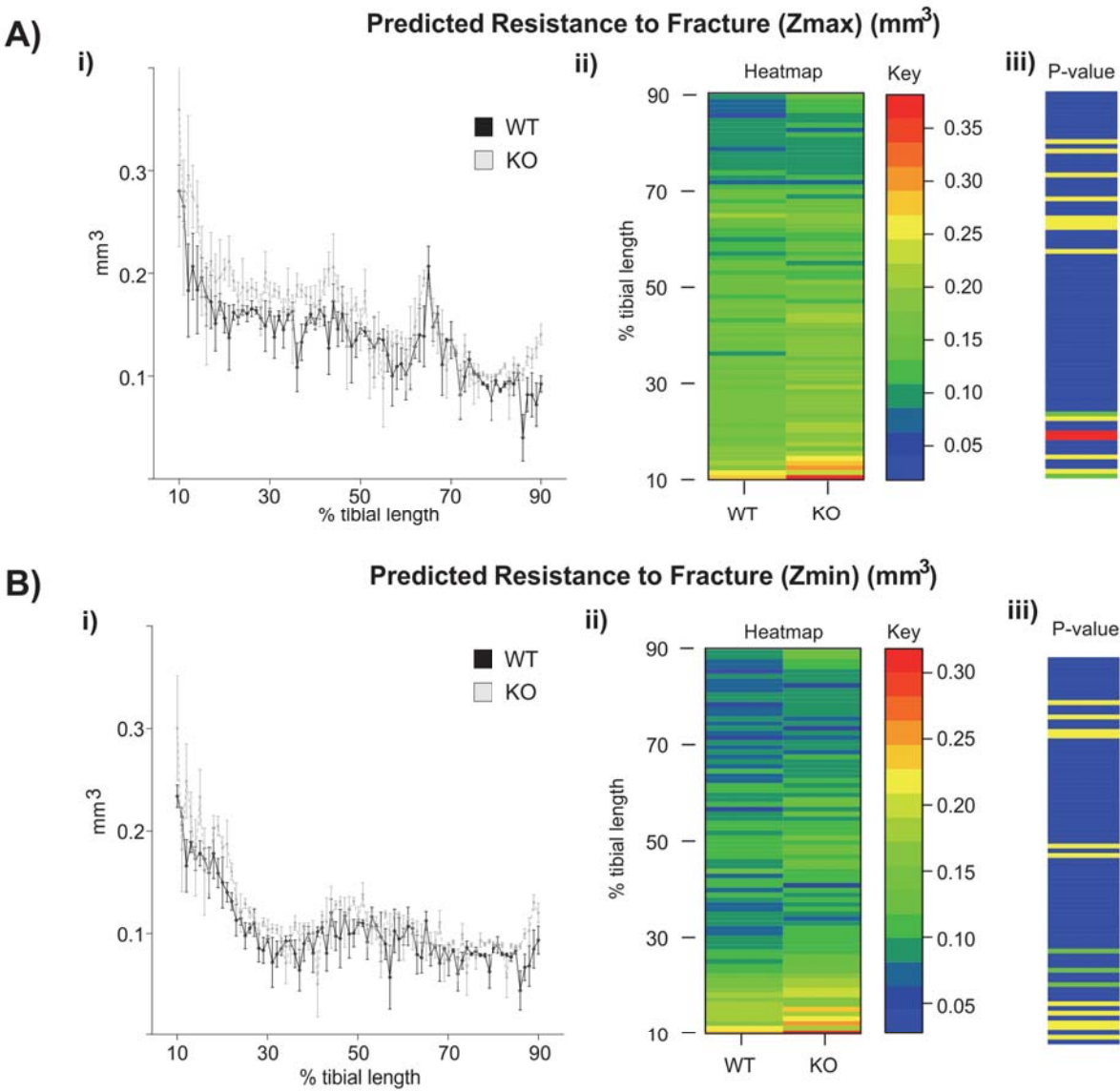

Supplement: Additional file 3: Figure S2. — Effects of loss of Lrrk2 on tibial cortical Zmax and Zmin. Images in Figures A and B show i) values for Zmax and Zmin, predicted resistance to fracture along the shortest and longest cross-sectional axes, respectively in female wild-type (WT) and Lrrk2 knockout (KO) mice; together with ii) the same data expressed as a graphical heat map along the tibial length; and iii) the points at which differences between genotypes for these parameters become statistically significant. (blue = n/s, yellow, p < 0.05, green p < 0.01, red, p < 0.001). (PDF 110 kb) [file 13024_2017_153_MOESM3_ESM.pdf]

Supplementary Figure 3

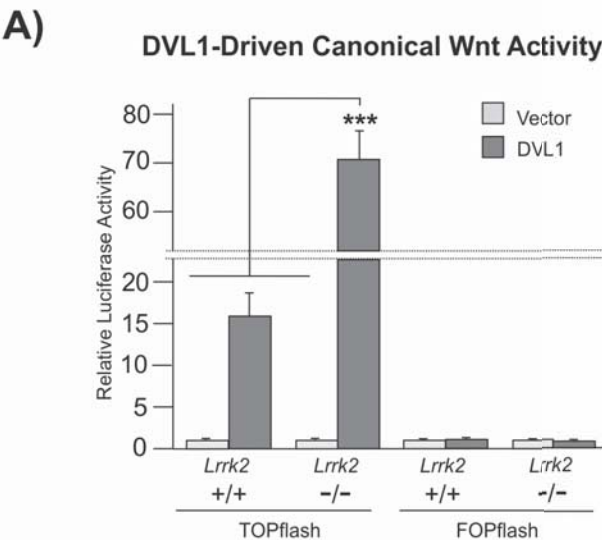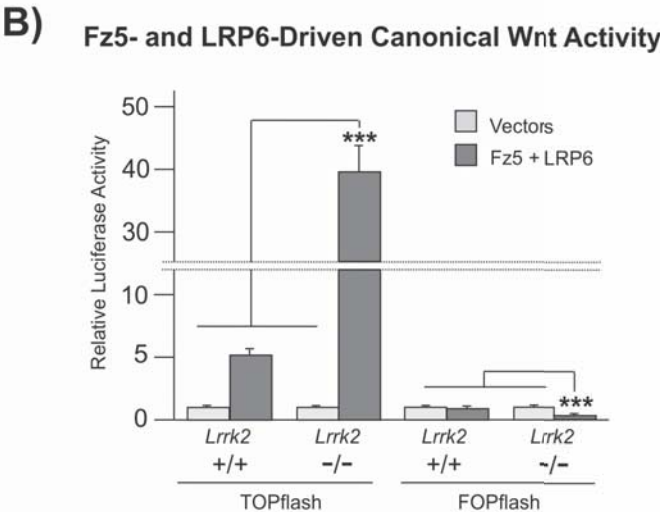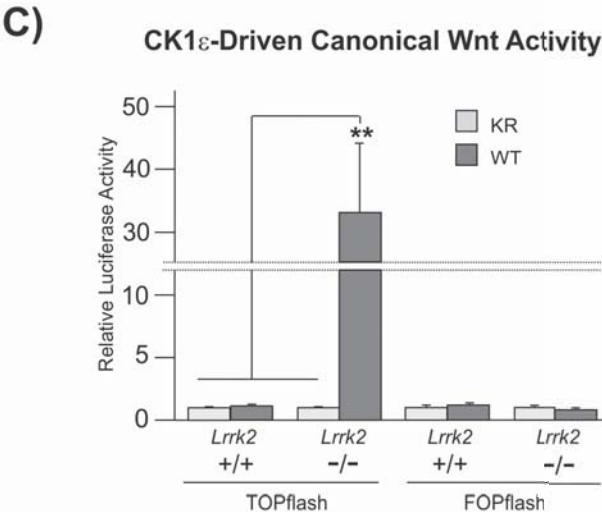

Supplement: Additional file 4: Figure S3. — Increased canonical Wnt activity in Lrrk2 knockout cells. A, B) Cells were transfected with TK-renilla and TOPflash or FOPflash in the presence of A) FLAG-DVl1 or C) GFP-FZD5 and HA-LRP6, or appropriate vector controls. A) 1-way ANOVA (n = 9; F = 146.199, p < 0.001) followed by Bonferroni post-hoc analysis revealed increased DVL1-driven TOPflash activity in Lrrk2 knockout cells (p < 0.001 versus all other conditions). No significant changes in FOPflash values were detected (n = 9; F = 2.668, p = 0.064). B) 1-way ANOVA (n = 9; F = 70.694, p < 0.001) followed by Bonferroni post-hoc analysis revealed increased GFP-FZD5/HA-LRP6-driven TOPflash activity in Lrrk2 knockout cells (p < 0.001 versus all other conditions). By contrast, the same treatment elicited a significant decrease in FOPflash values (1-way ANOVA: n = 6; F = 11.129, p = 0.001. Bonferroni post-hoc analysis p < 0.001). C) Wild-type and Lrrk2 MEFs were co-transfected with TK-renilla and TOPflash or FOPflash in the presence of active or inactive (KR) HA-tagged CK1ε. 1-way ANOVA (n = 9; F = 7.619, p = 0.001) followed by Bonferroni post-hoc analysis revealed increased CK1ε-driven TOPflash activity in Lrrk2 knockout cells (p < 0.01 versus all other conditions). No significant changes in FOPflash values were detected (n = 3; F = 1.535, p = 0.279). (PDF 90 kb) [file 13024_2017_153_MOESM4_ESM.pdf]

Supplementary Figure 5

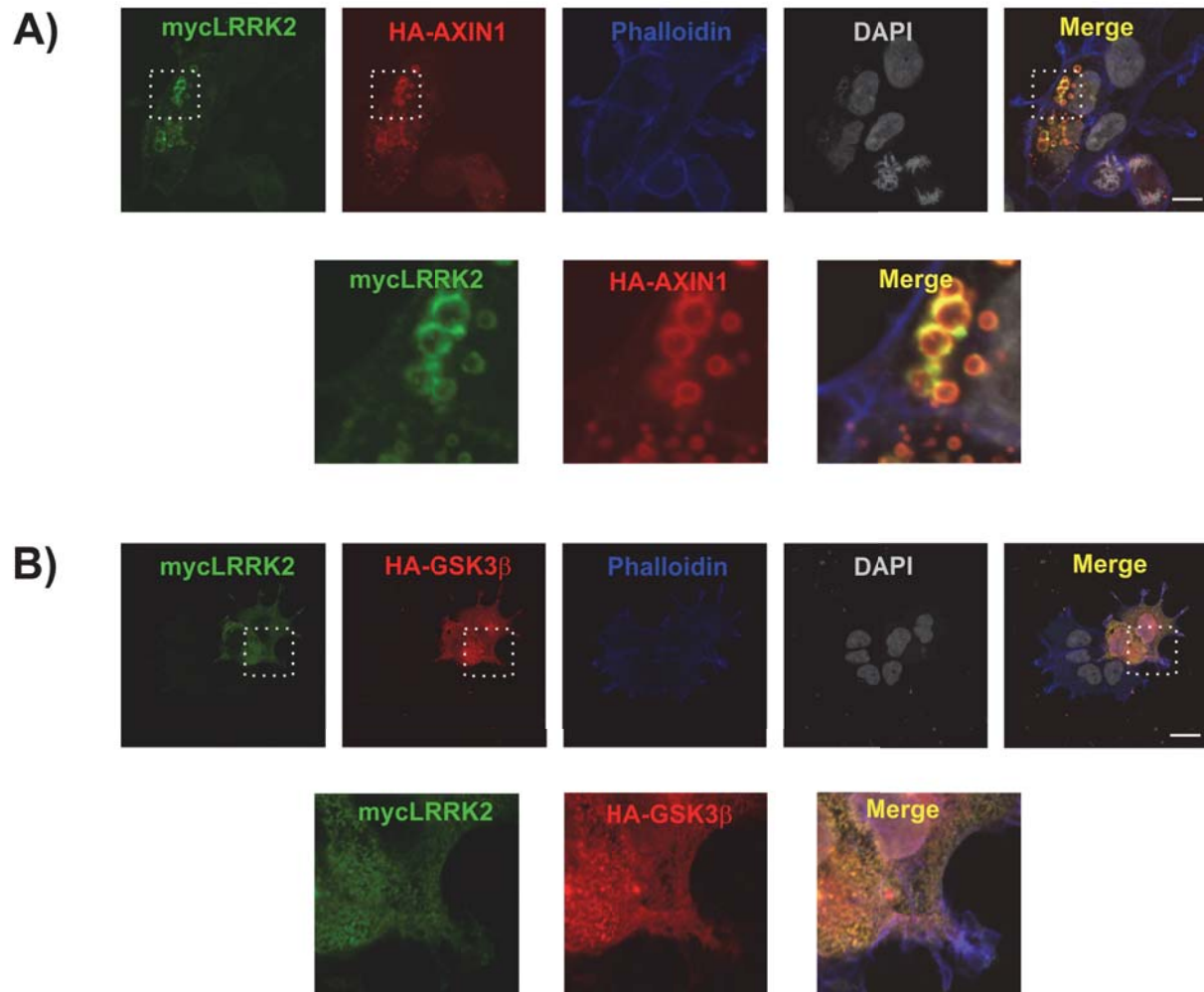

Supplement: Additional file 6: Figure S5. — Co-localisation between mycLRRK2 and HA-AXIN1, and mycLRRK2 and HA-GSK3β in HEK293 cells. A) Recruitment of mycLRRK2 (green) into polymers formed by HA-AXIN1 (red). Magnified images of a selected region are included. B) shows cytoplasmic co-localisation between mycLRRK2 (green) and HA-GSK3β (red), also with magnified images included. Note that in both experiments, counterstaining with phalloidin (blue) and DAPI (grey) were performed to visualise filamentous actin and chromosomal DNA respectively. The scale bar = 10 μm. (PDF 92 kb) [file 13024_2017_153_MOESM6_ESM.pdf]

Supplementary Figure 6

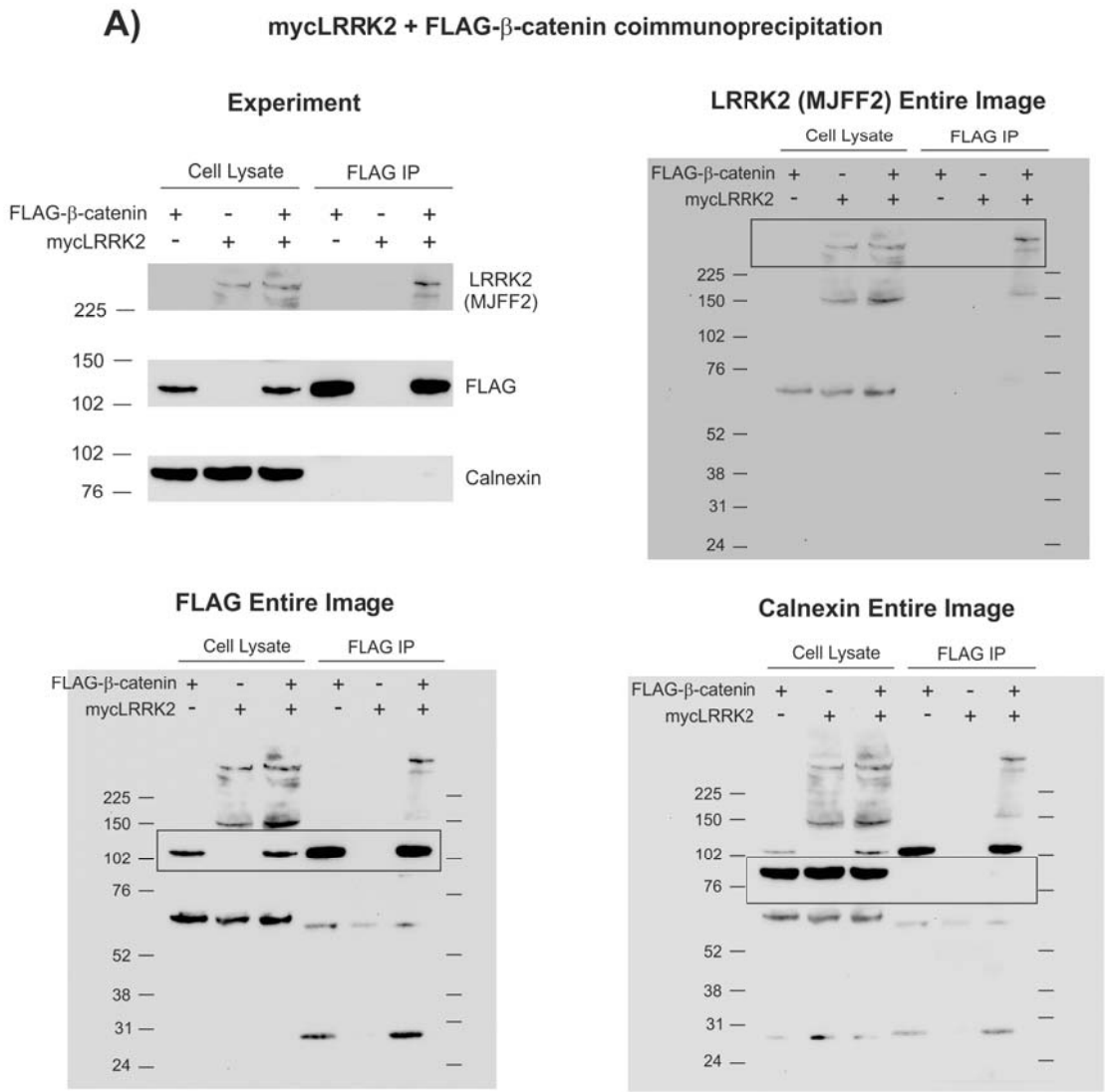

**B) mycLRRK2 + FLAG- $\beta$ -catenin Canonical Wnt Activity**

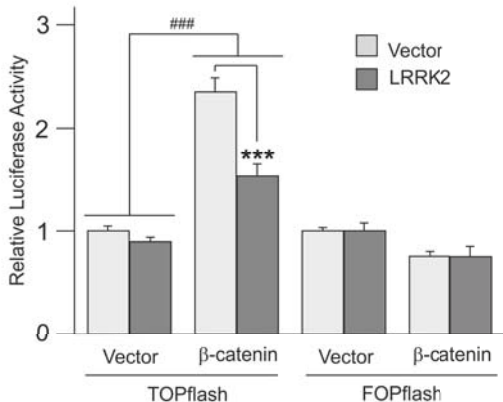

Supplement: Additional file 7: Figure S6. — LRRK2 interacts with β-catenin and represses β-catenin-driven TOPflash activity. A) HEK293 cells were co-transfected with FLAG-tagged β-catenin (lanes 1 and 4), myc-tagged LRRK2 (lanes 2 and 5), or myc-tagged LRRK2 and FLAG-tagged β-catenin (lanes 3 and 6) for 24 h prior to lysis. Lysates were immunoprecipitated with anti-FLAG antibodies and bound protein was resolved by Western blot (lanes 4–6), with the original lysates run alongside to confirm expression of transfected protein (lanes 1–3). Myc-tagged LRRK2 can clearly be seen in anti-FLAG immunoprecipitates from co-transfected cells (lane 6, upper panel). Full images of all blots are also shown. B) HEK293 cells were transfected with the TOPflash or FOPflash reporter plasmids with the indicated combinations of mycLRRK2, FLAG-β-catenin or appropriate control vectors. After 24 h lysates were taken and luciferase activity measured. 1-way ANOVA of TOPflash values revealed a significant effect of transfection on canonical Wnt activity (n = 9, F = 44.893, p < 0.001). Post-hoc Bonferroni testing showed significantly increased Wnt signalling caused by FLAG-β-catenin transfection in the presence or absence of mycLRRK2 co-transfection (p < 0.001 in both cases). Importantly however, co-transfected mycLRRK2 significantly weakened the TOPflash activation elicited by FLAG-β-catenin (p < 0.001). ANOVA of control FOPflash values also revealed significant effects of treatment, however Bonferroni post-hoc analysis found no significant differences in any pair-wise comparison. (PDF 129 kb) [file 13024_2017_153_MOESM7_ESM.pdf]

Supplementary Figure 7

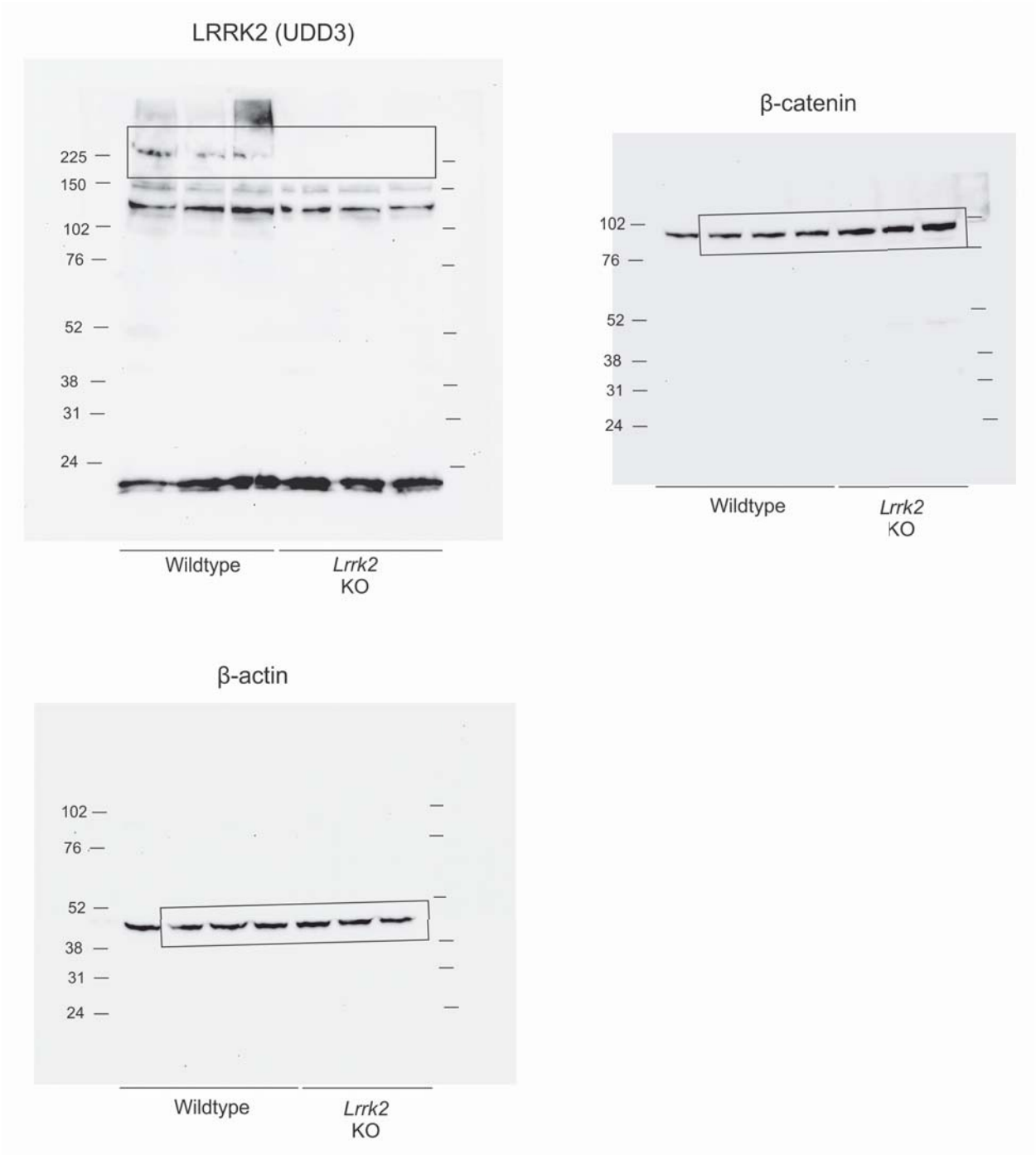

Supplement: Additional file 8: Figure S7. — Entire images of Western Blots shown in Fig. 4a. (PDF 70 kb) [file 13024_2017_153_MOESM8_ESM.pdf]

Supplementary Figure 8

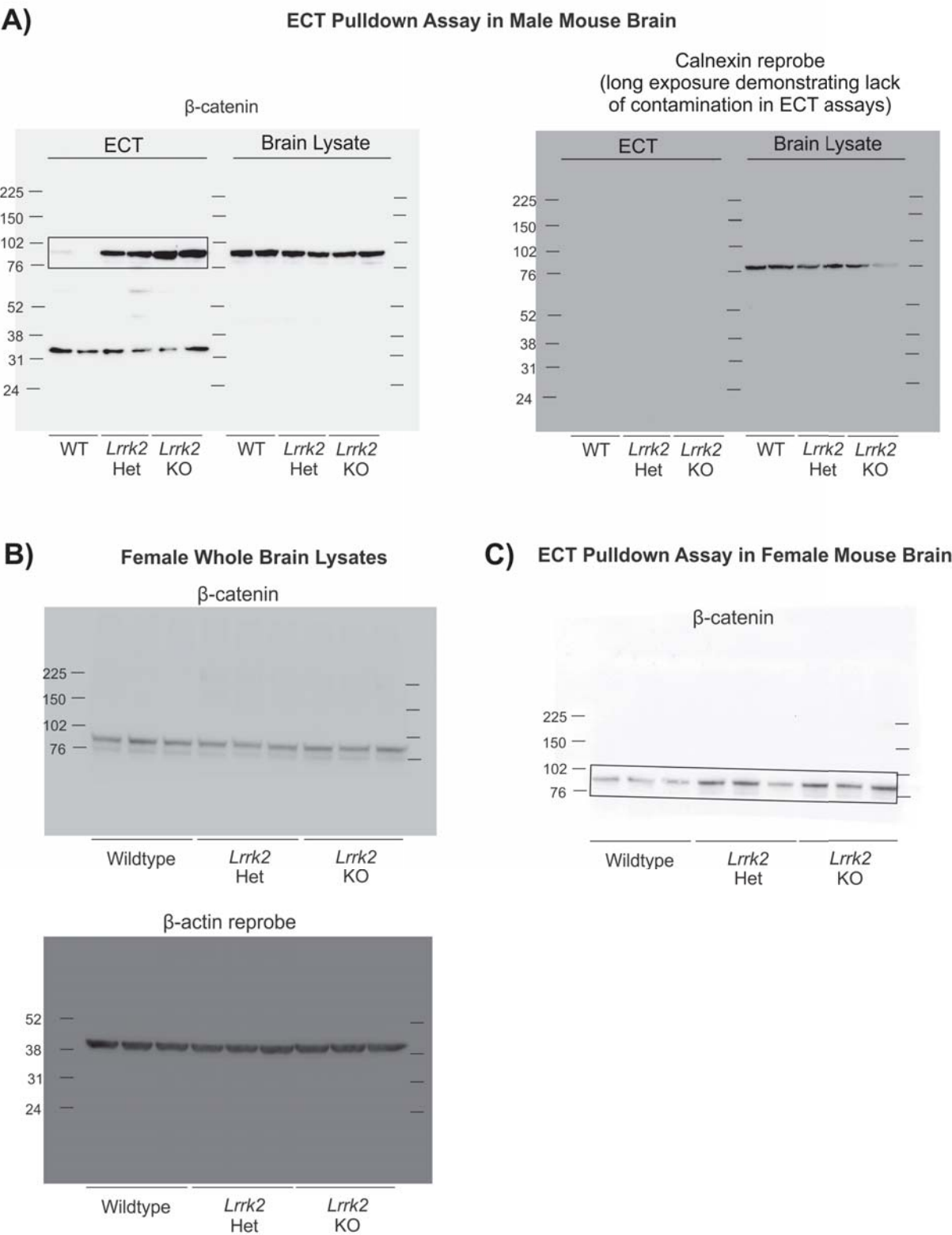

Supplement: Additional file 9: Figure S8. — Entire images of Western Blots shown in Fig. 4D and E and total β-catenin levels in aged female mouse brains A) Full blot from the image shown in Fig. 4d, plus a reprobe of the same membrane for calnexin to show that pulldowns are clean and this protein is only present in cell lysates. B) Western blotting shows no discernible differences between levels of total β-catenin in brains from aged female mice. C) Full blot from the image shown in Fig. 4e. (PDF 102 kb) [file 13024_2017_153_MOESM9_ESM.pdf]

## Supplementary Figure 9

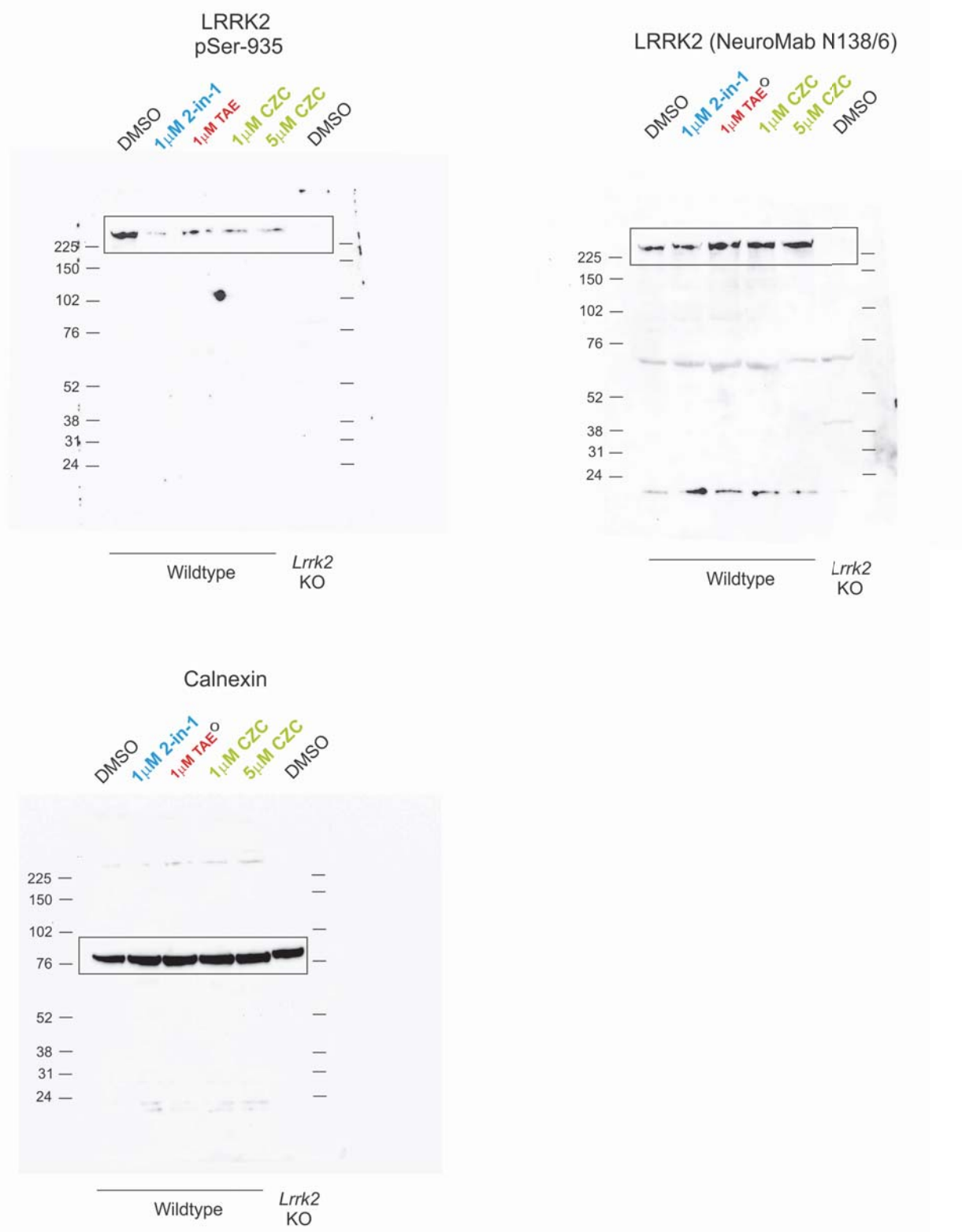

Supplement: Additional file 10: Figure S9. — Entire images of Western Blots shown in Fig. 5b. (PDF 80 kb) [file 13024_2017_153_MOESM10_ESM.pdf]
