# Supplementary material for: Pathogenic LRRK2 variants are gain-of-function mutations that enhance LRRK2-mediated repression of β-catenin signaling
Source: Mol Neurodegener. 2017 Jan 19;12:9. doi: 10.1186/s13024-017-0153-4 (PMC5248453; doi:10.1186/s13024-017-0153-4)

Supplementary Figure 4

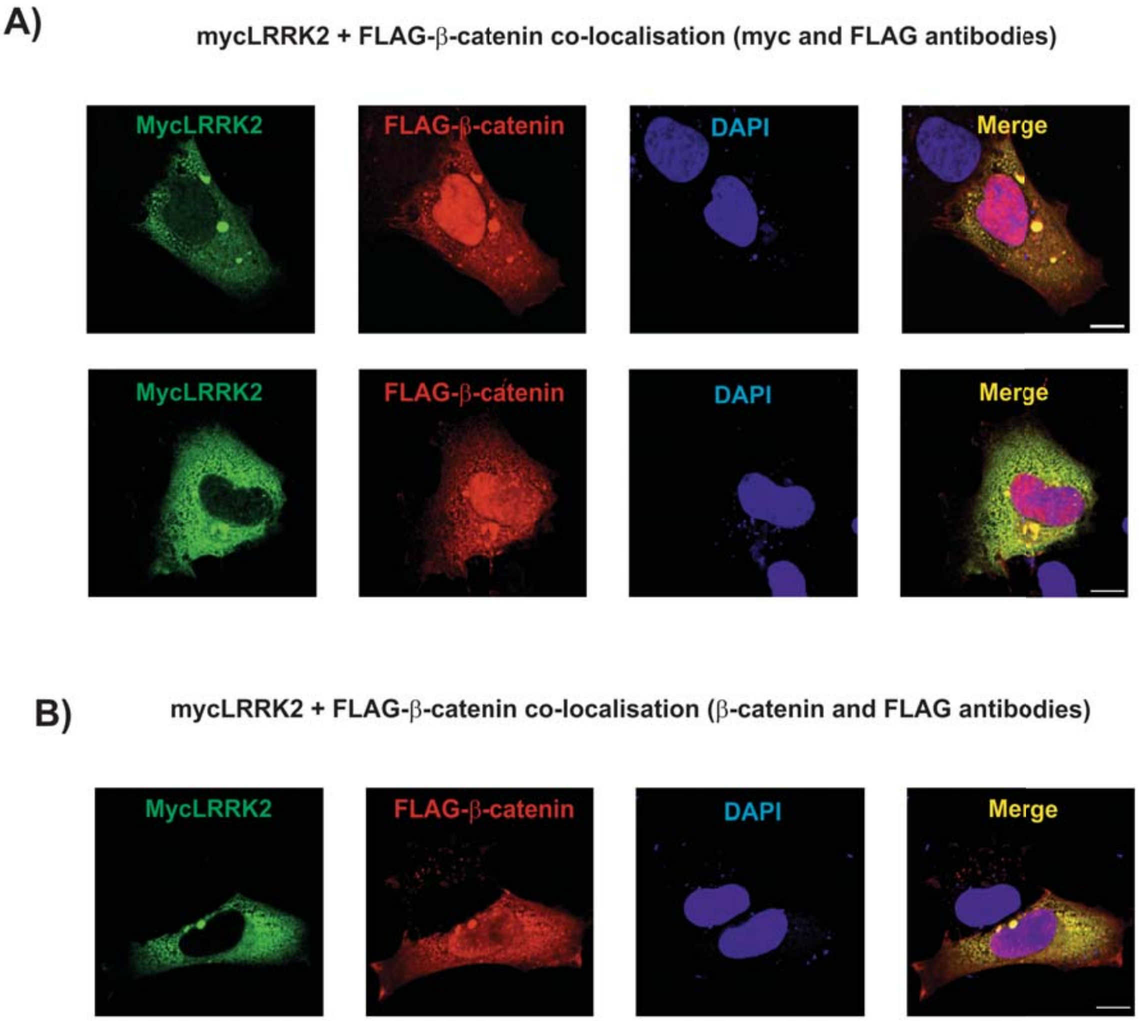

Supplement: Additional file 5: Figure S4. — Association of mycLRRK2 and FLAG-β-catenin in HEK293 cells 24 h post transfection, cells expressing myc-tagged LRRK2 and FLAG-tagged β-catenin were treated with MG132 for 1 h to allow β-catenin accumulation. The cells were subsequently fixed and stained with antibodies for A) myc (green) and FLAG (red), or B) myc (green) and β-catenin (red). DAPI staining (blue) was also performed to show cell nuclei. The right-hand panel shows an overlay of all channels – note the considerable co-localisation between LRRK2 and β-catenin in the cytoplasm. (PDF 102 kb) [file 13024_2017_153_MOESM5_ESM.pdf]
